# Toward a *Shigella* Vaccine: Opportunities and Challenges to Fight an Antimicrobial-Resistant Pathogen

**DOI:** 10.3390/ijms24054649

**Published:** 2023-02-28

**Authors:** Maria Michelina Raso, Vanessa Arato, Gianmarco Gasperini, Francesca Micoli

**Affiliations:** 1 GSK Vaccines Institute for Global Health (GVGH) S.r.l., Via Fiorentina 1, 53100 Siena, Italy; 2 GSK, Via Fiorentina 1, 53100 Siena, Italy

**Keywords:** *Shigella*, lipopolysaccharides, O-antigens, type III secretion system, vaccines, AMR

## Abstract

Shigellosis causes more than 200,000 deaths worldwide and most of this burden falls on Low- and Middle-Income Countries (LMICs), with a particular incidence in children under 5 years of age. In the last decades, *Shigella* has become even more worrisome because of the onset of antimicrobial-resistant strains (AMR). Indeed, the WHO has listed *Shigella* as one of the priority pathogens for the development of new interventions. To date, there are no broadly available vaccines against shigellosis, but several candidates are being evaluated in preclinical and clinical studies, bringing to light very important data and information. With the aim to facilitate the understanding of the state-of-the-art of *Shigella* vaccine development, here we report what is known about *Shigella* epidemiology and pathogenesis with a focus on virulence factors and potential antigens for vaccine development. We discuss immunity after natural infection and immunization. In addition, we highlight the main characteristics of the different technologies that have been applied for the development of a vaccine with broad protection against *Shigella*.

## 1. Introduction

**Figure 1:**
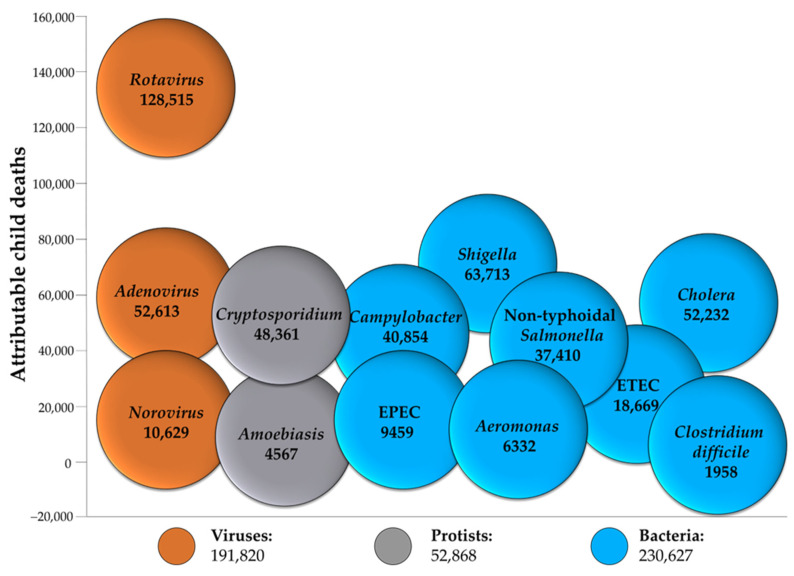
Major pathogens responsible for diarrhea in children under 5 years old. Each ball reports the number of deaths from diarrhea attributed to each pathogen in 2016 (data source: https://ourworldindata.org/diarrhoeal-diseases) (accessed on 23 January 2023). ETEC: Enterotoxigenic *E. coli*, EPEC: Enterotopathogenic *E. coli*.

According to recent global estimates, diarrheal diseases cause 1.6 million deaths (all ages) and are the third leading cause of death among children younger than 5 years behind pneumonia and preterm birth complications [1]. Diarrheal diseases mainly affect low- and middle-income countries (LMICs), where accessibility to clean water, good nutrition, sustained sanitation, and healthcare are restricted and represent the leading risk factors of the disease. Among children under 5 years old, the three etiologies responsible for most of the deaths are Rotavirus, *Shigella* spp., and *Cryptosporidium* spp. [2], respectively, from viruses, bacteria, and protists (Figure 1).

Here, we focus our attention on *Shigella*, the leading bacterial cause of diarrheal disease, and its virulence factors. Recently, the onset of antimicrobial resistance (AMR) has increasingly been associated with this pathogen [3], making the development of vaccines against *Shigella* an even greater global health priority. To date, no vaccines are widely available but different approaches are being evaluated in the clinic for the development of broadly protective vaccines. Here, we describe potential antigens that have been proposed to fight shigellosis and highlight the pros and cons of the different technologies applied for the development of a vaccine.

## 2. Shigellosis

### 2.1. Shigella Bacteria

*Shigella* are facultative anaerobic, non-motile, non-sporulating, rod-shaped, Gram-negative bacteria belonging to the family of *Enterobacteriaceae*. The bacteria are facultative intracellular pathogens that show a high specificity for the human host in which they cause shigellosis, commonly known as bacillary dysentery [4]. At the end of the 19th century, during an epidemic in Japan associated with high mortality, the microbiologist Kiyoshi Shiga isolated and characterized the bacteria causing such disease, which was later named *Shigella* [5].

The current classification divides the genus *Shigella* into four species based on serological typing: *S. dysenteriae*, *S. boydii*, *S. flexneri*, and *S. sonnei* [6]. These species are further distinguished into serotypes and subserotypes based on the specificity of the saccharide repeating units that form the O-Antigen (OAg) portion of lipopolysaccharides (LPSs). At present, the species *S. dysenteriae* is known to comprise 14 serotypes, *S. boydii* comprises 19 serotypes, *S. flexneri* comprises 15 serotypes and sub-serotypes, and *S. sonnei* comprises only one serotype [7]. Through different methodologies, it has been established that *Shigella* spp. belong to *E. coli* species [8,9,10]. Based on whole-genome sequencing, *E. coli* and all four *Shigella* species were found to share a common DNA backbone of approximately 3.9 Mb, interrupted by sequences specific to *E. coli* or *Shigella*. Comparative genomics indicate that entero-invasive *E. coli* (EIEC) is genetically more related to *Shigella* than to non-invasive *E. coli* [11,12]. *E. coli* and *Shigella* genomes mainly differ in the presence of many insertion sequence (IS) elements in *Shigella*, with a very dynamic genome [13,14]. The easy acquisition and loss of genes promote the success of *Shigella* as a pathogen because fast genetic adaptation, plus the acquisition of a single-copy virulence plasmid, allows bacteria to survive in different circumstances in the host [15,16].

To invade, replicate, and disseminate throughout the intestinal epithelium *Shigella* uses a range of bacterial effector proteins that are encoded by a large 220 kb plasmid called virulence plasmid or invasion plasmid (pINV), common to all *Shigella* species [17]. The pINV is composed of a 30 kb pathogenicity island (PAI) that encodes the type III secretion system (T3SS) and other virulence factors involved in adhesion and actin-mediated motility, such as IcsA/VirG. In the case of *S. sonnei* only, the pINV also encodes the serotype-specific OAg biosynthesis locus. It has been shown that the pINV is less stable in *S. sonnei* than in *S. flexneri*, especially at environmental temperatures. The pINV maintenance depends on a toxin–antitoxin (TA) system, consisting of a toxin and a related antitoxin that blocks its activity. Such a TA system is present in pINV from *S. flexneri* but not from *S. sonnei* [18].

### 2.2. Transmission and Pathogenesis

The transmission route for *Shigella* infections is fecal–oral, usually through close contact between people or through ingestion of contaminated food or water [19]. 

**Figure 2:**
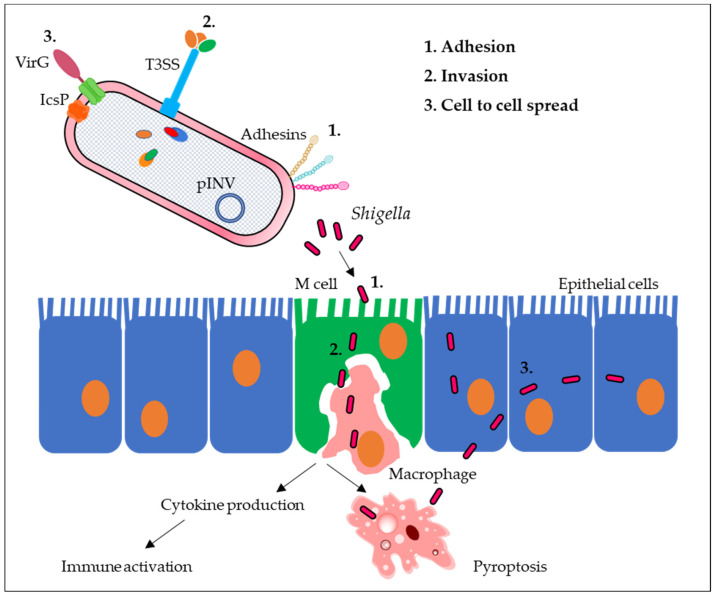
Infectious cycle of *Shigella*.

Clinical symptoms may be mild, such as watery diarrhea, but the disease can evolve into severe inflammatory bacillary dysentery with abdominal cramps, fever, nausea, anorexia, dehydration, and stools characterized by the presence of blood and mucus. Surprisingly, the inoculum size necessary for *Shigella* infection is as low as 10–100 bacteria [20]. The infection is non-systemic and enterically invasive, leading to the destruction of the colonic epithelium. Following oral ingestion, *Shigella*, after surviving the stomach’s acidic environment and the competitive gut microbiota, arrives at the terminal ileum, colon, and rectum where it permeates the mucous layer. *Shigella* needs to survive multiple barriers to arrive at the epithelial surface, including microbiota-mediated colonization resistance, locally-produced antimicrobial peptides, and mucinases [21]. To establish an infection, *Shigella* transits across the colonic epithelial layer through M cells allowing translocation of bacteria across the epithelial barrier without damaging these cells [22] (Figure 2) and efficiently invades colonic epithelial cells from the basolateral side. Bacterial spread from cell to cell is the main step towards a severe inflammatory response [23] that leads to epithelium disruption, allowing the translocation of further bacteria [24].

Upon internalization, *Shigella* replicates and disseminates within the mucosal epithelium and causes relevant damage to the cellular cytoskeleton. The bacillus can be engulfed by macrophages and dendritic cells, which consequently undergo pyroptosis. The consequent host innate immune response activates the recruitment of neutrophils and the production of inflammatory cytokines, causing epithelial abscesses, ulcerations, and demolition, that further enhance *Shigella* invasion via a disrupted epithelial barrier [7]. *Shigella’*s ability to endure intracellularly and avoid phagocytic killing depends on T3SS effectors that reduce inflammatory responses by inhibiting the host cell’s pro-inflammatory signaling pathways and cytokine production. Examples of these effectors are OspG, which impairs NF-κB activation, and IpaH9.8, which is translocated into the nucleus of epithelial cells and dampens the expression of pro-inflammatory cytokines and chemokines [25]. These mechanisms together with serotype diversity affect the development of long-term, broadly effective protection [26,27].

*S. flexneri* (2a and 5a) strains have been the model organisms used to characterize *Shigella* virulence mechanisms [4]. However, each *Shigella* species and subspecies appear to have different virulence mechanisms and so deserve a specific analysis [23].

### 2.3. Epidemiology and Medical Needs

**Figure 3:**
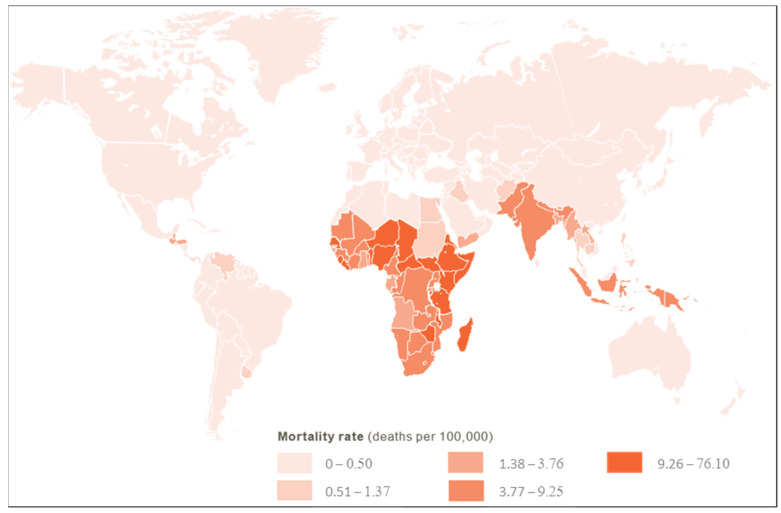
*Shigella* diarrhea mortality rate (deaths per 100,000). Data from [28].

*Shigella* is a top cause of Moderate to Severe Diarrhea (MSD) throughout the world. Estimates report approximately 270 million cases with 212,438 total deaths per year with 30% in children younger than 5 years, especially in LMICs (Figure 3), and 2–7 cases of *Shigella* per 100 child-years require clinical care [28]. The incidence of *Shigella* infections is very low during the first six months of life, most probably because of maternal antibody titers and the low direct interaction with the external environment. Incidence increases after this age, peaking at 12–23 months and moderately decreasing afterward [29].

Shigellosis rates in high-income countries (HICs) are much lower, at around 0.01 per 100 person-years among all ages and 0.02 per 100 person-years among children [30].

Data from the Global Enteric Multicenter Study (GEMS), a 3-year, prospective, age-stratified, case/control study focused on the investigation of the population-based burden, etiology, and adverse clinical consequences of acute MSD among children younger than 5 years living in sub-Saharan Africa and South Asia, established *Shigella*, identified by culture, as the most common etiology in children aged 24–59 months and the second most widespread etiological agent among children aged 12–23 months [31]. Among 1130 *Shigella* isolates, *Shigella dysenteriae* and *S. boydii* accounted for 5.0% and 5.4%, respectively, while *S. flexneri* accounted for 65.9% and *S. sonnei* accounted for 23.7%. Among all *S. flexneri* serotypes, five were predominant (that comprised 89.4% of *S. flexneri*) and included *S. flexneri* 2a, *S. flexneri* 6, *S. flexneri* 3a, *S. flexneri* 2b, and *S. flexneri* 1b [32].

Upon re-analysis of GEMS samples with quantitative PCR, the attributable incidence of *Shigella* more than doubled [29]. *Shigella* resulted as the major pathogen associated with dysentery (attributable fraction 63.8%) and was also the second most common pathogen associated with watery diarrhea (attributable fraction 12.9%) [29]. These findings indicate that the burden of shigellosis as defined using culture-based diagnostics is probably underestimated. The current worldwide epidemiological burden of shigellosis is mainly attributed to two species, *S. flexneri* and *S. sonnei*, which were traditionally associated with developing and developed regions, respectively [33,34,35]. Nevertheless, recent evidence indicates the emergence of *S. sonnei* in economically transitional states, effectively replacing *S. flexneri* and becoming the predominant shigellosis etiology [36]. This species replacement phenomenon has been documented several times in many countries in Asia, such as Vietnam [37], Thailand [38], and Bangladesh [39]. The reasons behind this serotype replacement are still under investigation; however, one hypothesis is that the competitive advantage of *S. sonnei* over *S. flexneri* is due to the exceptional ability of the former to acquire antimicrobial resistance genes from both commensal and pathogenic bacteria, especially in areas where the use of antimicrobials is poorly regulated. Furthermore, it has been reported that *S. sonnei* has an increased ability to grow successfully within *Acanthamoeba* present in public water supplies, which acting as host, allows the bacterium to persist in adverse environmental conditions [36,40].

The prevalence of *Shigella* species and serotypes changes from country to country, over time, and with economic status. Limited epidemiological data are available for *S. boydii*, which is uncommon in many regions of the world, with exceptions being the Indian subcontinent [41] and Latin America [42,43]. In India, a heterogeneous distribution of *Shigella* species and serotypes [44] has been reported between 2001 and 2003, with *S. flexneri* being the most common (45%) followed by *S. dysenteriae* (29.4%), *S. boydii* (14.7%), and *S. sonnei* (10.2%). A dramatic change was observed in the same study conducted in South India between 2003 and 2006, where *S. flexneri* remained the most prevalent (45%) followed by *S. sonnei* (31%), *S. boydii* (15%), and *S. dysenteriae* (8%). In Andaman and Nicobar Islands, the *S. flexneri* 2a serotype was identified to be predominant [45]. A heterogeneous distribution of *Shigella* serotypes was found in cases from urban and rural areas of Bangladesh [46]. *S. dysenteriae*, specifically *S. dysenteriae* 1, caused many cases of fatal dysentery epidemics since its first isolation in 18,975. However, this species is rarely isolated in current surveillance. Improvements in sanitation and anti-microbial access have been proposed to have caused a decline in illness and death related to *Shigella* spp., including *S. dysenteriae* [47,48].

A unique epidemiological niche for *Shigella* emerged in the 1970s as a sexually transmitted infection among men who have sex with men (MSM). In 2009, serotype *S. flexneri* 3a appeared in England and Wales among MSM and rapidly spread within the MSM population to regions considered at low risk for shigellosis, causing an epidemic. 

Around 536 million people travel to LMICs annually and 10–40% acquire diarrhea. It has been estimated using faecal cultures that 2–9% of travelers’ diarrhea is attributable to *Shigella* [49]. Repercussions of shigellosis during travel are the introduction of antimicrobial-resistant *Shigella* into new populations [50] and outbreaks in tourist groups and military settings. An updated review, that includes publications between January 1990 and June 2015, reveals that travelers’ diarrhea continues to be a major medical concern for deployed US military personnel and other long-term travelers, with an incidence of over 30 cases per 100 person-months [51]. Furthermore, this phenomenon increases the possibility of reinfections in areas where other serotypes predominate.

Controlling the burden of *Shigella* is challenging for many reasons. First, *Shigella* needs a low infectious dose for transmission through the fecal–oral route, and the possibility of reinfection due to the variety of *Shigella* species and serotypes coexisting in the same areas is high. In addition, the need for antibiotic therapy, not just oral rehydration, further complicates treatment and paves the way for the emergence of multidrug-resistant strains of *Shigella*. This threatens the administration of effective, affordable treatments and highlights the importance of infection prevention [52,53].

### 2.4. Antibiotic Resistance of Shigella

*Shigella* has been listed by the WHO among those pathogens for which the development of new interventions is a priority [54]. The use of antibiotics for the treatment of common bacterial infections plays an important role in reducing the burden of the disease. Nevertheless, antibiotic overuse to treat diarrhea increases antibiotic resistance [55]. *Shigella* spp. is resistant to most antibiotics, and drug treatment is expensive, time-consuming, and sometimes problematic, especially in areas where medical care is limited [44,56]. 

**Figure 4:**
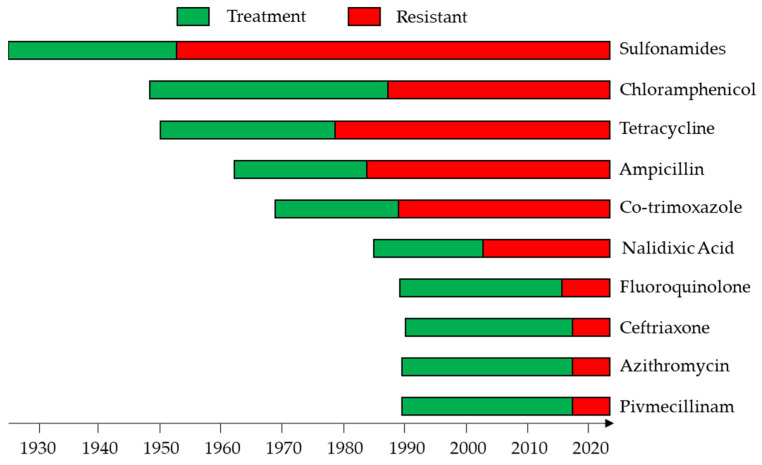
Global drug treatment against *Shigella* infection over the years and increase in antibiotic resistance developed to these drugs.

After sulfanilylguanidine was shown to be bacteriostatic against *Shigellae* [57] in the late 1930s (Figure 4), sulfonamides became the treatment of choice for shigellosis [58]. Favorable clinical and bacteriological results were achieved also with the use of oral chloramphenicol [59]. Tetracycline was used routinely in the 1950s and 1960s, but it was phased out because of widespread resistance and the side-effect on the discoloration of teeth in children [60]. *Shigella* developed resistance to all of these drugs, so the treatment shifted to ampicillin and co-trimoxazole. However, treatment recommendations were again changed to nalidixic acid because *Shigella* developed resistance to the former drugs. Later, when resistance capacity to nalidixic acid occurred [48], fluoroquinolones were introduced. However, fluoroquinolone-resistant strains have been isolated from various sources [61]. At the moment, the WHO recommends ceftriaxone, azithromycin, and pivmecillinam for treatment of infection by fluoroquinolone-resistant *Shigella* spp. [44]. Unfortunately, ceftriaxone-resistant and azithromycin-resistant strains have already been isolated in some places [62].

About half of the *Shigella* strains are now resistant to several drugs [63] in many areas of the world, and this has obviously become a matter of huge concern. Bacteria can exploit several antibiotic-resistance mechanisms, among them are the use of efflux pumps to extrude the drugs, the change in membrane permeability, and the expression of enzymes able to modify and inactivate the molecules [56,64,65,66,67]. For example, β-Lactam antibiotics, a class of antibiotics characterized by a β-lactam ring in their molecular structure, inhibit cell wall biosynthesis by acting on penicillin-binding proteins. Furthermore, the mutation or absence of porin in the outer membrane of Gram-negative bacteria such as *Shigella* spp. decrease the penetration of β-lactams, such as aztreonam and dianionic moxalactam, and also the susceptibility of hydrophilic antibiotics, such as penicillin and piperacillin [64,68]. Specifically, resistance to β-lactam antibiotics is associated with modifications on OmpF and OmpC and to OmpR, which act as a transcriptional regulator [64]. In addition, LPS has been recognized as an important key player in antibiotic resistance as it is essential for the assembly of trimeric PhoE porin, confers colicin E2 resistance in *S. flexneri* strains [69], and has been linked with the rise in resistance toward imipenem in *S. dysenteriae* [70].

There is a pressing need for the development of effective strategies to fight AMR *Shigella* infections [71], and some therapeutic approaches have been proposed including the use of natural products, nanoparticles (NPs), and phage therapy in addition to vaccine strategies [72]. Studies have shown the efficacy of natural small molecules produced by microbes, plants, and animals for treating *Shigella* infections. Probiotics have been suggested as prophylaxis for antibiotic-induced diarrhea (e.g., for travelers) and are also an alternative therapeutic choice for the treatment of gastroenteritis infections [73]. NPs are also gaining more attention as they have shown a broad spectrum of antibacterial activity against pathogenic bacteria, due to their bactericidal characteristics [74]. In particular, iron oxide nanoparticles (IONPs) destroy bacterial targets by affecting membrane cell integrity and by generating free oxygen radicals. Bacteriophages kill bacteria through a lysis mechanism. In addition, phage therapy has shown to be advantageous, especially for its specificity in targeting bacterial pathogens without effecting the normal human microflora. Phages replicate at the infection site, and they have a strong bactericidal effect on antibiotic-resistant strains and fewer side effects than other therapies. However, phages are self-limiting as they persist at a shallow level on target sites [75].

Vaccines can play a major role in fighting AMR. They are used prophylactically, decreasing the number of infectious disease cases, and thus antibiotic use and the emergence and spread of AMR [76]. Different candidate vaccines have been proposed to prevent infection by *Shigella* spp., most of which are currently being evaluated for safety and immunogenicity in clinical trials. 

## 3. *Shigella* Virulence Factors

### 3.1. Structure and Role of Shigella O-Antigens

**Figure 5:**
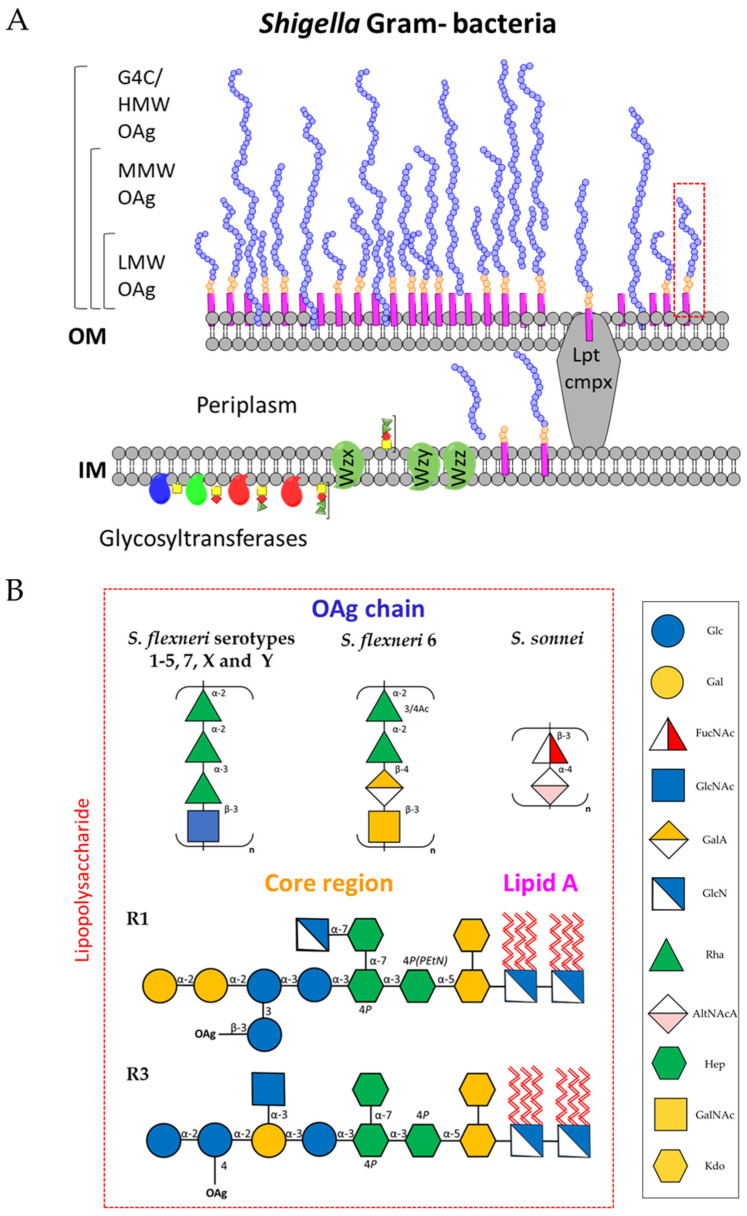
Biosynthesis of LPS moieties expressed on the membrane surface of *Shigella* bacteria (**A**). Structures of *Shigella* O-antigens and core regions (**B**). KDO: 3-deoxy-D-manno-2-octulosonic acid; Hep: L-glycero-D-manno-heptose; GlcN: glucosamine; GlcNAc: N-Acetylglucosamine; Rha: Rhamnose; GalA: galacturonic acid; GalNAc: N-Acetylgalactosamine; AltNAcA: N-Acetyl-amino altruronic acid; FucNAc: N-Acetyl-L-fucosamine; cmpx: complex; G4C: group 4 capsule; HMW: high molecular weight; MMW: medium molecular weight; LMW: low molecular weight.

Here, we focus on the structures and role of OAg from *S. sonnei* and *S. flexneri*, which are the most epidemiologically relevant *Shigella* serotypes. Gram-negative bacteria, such as *Shigella*, display LPS molecules on the cell surface (Figure 5). The LPS consists of a polysaccharide chain of repeating units, called OAg, linked to a core oligosaccharide. The core is anchored to the membrane through a lipid A moiety.

In *Shigella*, as in all enterobacteria, the conserved inner-core part is located closer to lipid A and consists of two residues of 3-deoxy-D-manno-2-octulosonic acid (KDO) and three residues of L-glycero-D-manno-heptose (Hep). Interestingly, the *Shigella* inner-core is identical to that of *E. coli* and belongs to the so-called *Salmonella* type. This is characterized by phosphorylation or glycosylation by α-GlcN or α-GlcNAc of the heptose residues. Moreover, the phosphate group on Hep^I^ is often non-stoichiometrically phosphorylated by 2-aminoethylphosphate (PEtN), forming P-PEtN. The outer-core part (connecting the inner-core to the OAg) has instead a less conserved structure. However, R1 outer-core types have been found in *S. sonnei* and *S. flexneri* 6, while R3 outer-core types have been found in all *S. flexneri* serotypes [77] (Figure 5).

All *S. flexneri* serotypes 1–5, 7, X, and Y, but not serotype 6, share a linear tetrasaccharide backbone consisting of the following OAg repeating unit: (2)-α-L-Rha*p*^III^-(1→2)-α-L-Rha*p*^II^-(1→3)-α-L-Rha*p*^I^-(1→3)-β-D-Glc*p*NAc-(1→)_n_, variably O-acetylated and/or glucosylated. Phosphorylation with PEtN has been classified into subtypes 4av, Xv, Yv, and Yv1 and described as “variant” subtypes by adding the letter “v” to the names of the corresponding PetN-negative subtypes [78]. *S. flexneri* serotype 6 is instead characterized by a linear polysaccharide backbone (2)-α-L-Rha*p*^III^-(1→2)-α-L-Rha*p*^II^-(1→4)-β-D-Gal*p*A-(*1*→3)-β-D-Gal*p*N*A*c-(1→)n, where the first Rha^III^ is variably O-acetylated in position 3 or 4 [79,80]. *S. sonnei* OAg repeat has a quite different structure, constituted of (4)-α-L-AltNAcA-(1→3)-β-FucNAc4N-(1→)n [81] (Figure 5).

The OAg biosynthesis depends on the Wzx/Wzy pathway. The inner membrane (IM) represents the site of glycan biosynthesis, where the Wzx proteins allow each single repeating unit produced by the glycosyltransferases to translocate from the cytoplasmic to the periplasmic side. After transportation of the repeating unit to the periplasm by Wzx, these are polymerized by Wzy into OAg chains, whose length is regulated by the Wzz family of proteins. The OAg is then ligated to a sugar residue of the core oligosaccharide by the WaaL ligase to form the complete LPS, which is then transported to the outer membrane (OM) by the Lpt complex. The OAg repeating units can also be polymerized into capsular polysaccharides (group 4 capsule, G4C) if, in addition to the wzx–wzy cluster, an additional G4C operon is present [82,83,84]. Formation of G4C has been reported for *S. sonnei* and *S. flexneri* 6 strains [83,84,85].

LPS molecules on the bacterial surface have a significant role in the pathogenesis of *Shigella*, including the protection of the bacteria from the lytic action of serum complement and the promotion of adhesion and internalization of bacteria to intestinal epithelial cells [86]. The diversity of the *S. flexneri* OAg is considered an important virulence factor that enhances the survival of pathogens. In addition, modifications such as serotype-specific glucosylation promote invasion of *S. flexneri* into host cells [87].

### 3.2. Structure and Role of T3SS Proteins

The weaponry that *Shigella* employs for tissue invasion and an intracellular lifestyle is encoded by a single DNA fragment of approximately 30 kb placed in the virulence plasmid [88]. The central element of this machinery is the type III secretion system (T3SS) [14] that forms a macromolecular needle-like structure across the membrane of the target eukaryotic cell and enables the bacteria to translocate at least a set of different effector proteins from their bactplasm directly into the eukaryotic host cells. Once injected, these virulence factors affect host cell function, support infection, and help the bacteria to evade host cell immune responses [4,21,89,90].

The complexity of the T3SS structure and composition has been deeply investigated over the years [91]. Briefly, in the structure of T3SS, it is possible to identify three elements: a cytosolic complex that forms the so-called “sorting platform”, the transmembrane basal body crossing the inner and the outer membranes, and the extracellular needle with the tip complex that protrudes on the bacterial surface. At the distal end, the tip is composed of IpaB, IpaC, and IpaD [92]. IpaD facilitates the assembly of IpaB and IpaC into the needle, and IpaB and IpaC are hydrophobic proteins able to form a pore in eukaryotic membranes, thus allowing for effectors delivery [92,93]. Membrane insertion and T3SS activity are promoted by the interaction of IpaB with cholesterol in the host membrane [94,95]. The needle has a helical symmetry resulting from the homopolymerization of approximately 100 MxiH subunits, and it has been proposed to have a role in propagating secretion signals [96]. Indeed, specific amino acid changes in MxiH are implicated in controlling the amount and type of the secreted substrates. However, it is still not clear how such signals would be received at the tip of the MxiH assembly.

The needle is inserted in the basal body consisting of an outer ring (composed of the secretin MxiD) that contacts the inner ring (composed of MxiG). Just below the basal body, there is the sorting platform with its major component Spa33 [97]. Spa33 interacts with the hub of the sorting platform that is formed by the stalk Spa13 [98,99] and Spa47, and this complex has been suggested to bind chaperones to help guide the secretion cargo, including IpaB/IpaC [100]. IpaB has been shown to be the first translocator protein recruited to the needle tip.

Multiple physiological factors (such as cell contact, media composition, serum, temperature, and pH) as well as artificial inducers (such as the Congo Red dye) have been shown to regulate the expression of T3SS genes through transcriptional activators belonging to the AraC family [101,102].

The T3SS has uniquely evolved to upregulate *Shigella* virulence in response to environmental levels of the bile salt deoxycholate (DOC), essential for proper dietary fat solubilization and intestinal absorption [103]. As other enteric pathogens, *Shigella* encounter high concentrations of DOC in the small intestine and in the colon. Bile enhances the expression of the *Shigella* T3SS effectors ospE1/ospE2 as adhesins [104]. Furthermore, the T3SS tip protein IpaD binds DOC and undergoes conformational changes supporting the recruitment of IpaB [105,106,107]. Upon contact with host membrane components, IpaC is recruited to the tip of the T3SS, completing the assembly of the translocon pore in the host cell membrane and promoting injection of effectors into the host cell and pathogen entry [95].

### 3.3. Role of Other Virulence Factors

The ability to adhere to host cells is extremely important for intracellular pathogens, and adhesins are a diverse class of virulence factors [108]. Few studies have examined the molecular mechanisms of *Shigella* adhesion. *Shigella* can spread intercellularly using actin-based motility (ABM), a process in which the host cell cytoskeleton is used by the bacterium to spread from cell to cell and to avoid extracellular immune defenses [109]. ABM is mediated by the autotransporter protein IcsA (VirG), whose secretion is independent of T3SS. IcsA is located in the outer membrane with asymmetric conformation through the action of several proteins such as DegP, Skp, and SurA [110,111], and newly formed IcsA is preferentially incorporated into the old pole of the bacterium. Polar localization of IcsA is critical to control the unidirectional movement of the bacterium and is maintained by IcsP, OmpA, the chaperon DnaK, and the enzymes YidC and Apyrase [112,113,114,115,116]. In particular, IcsP is an outer membrane protease that cleaves IcsA, and both of them are encoded by the pINV. *S. flexneri* IcsP mutants show reduced amounts of secreted IcsA passenger domain in culture supernatants and display IcsA across the entire cell surface. The expression of IcsP is finely tuned by the Fur/RyhB regulatory pathway in response to the low concentrations of free iron, a condition encountered within colonic epithelial cells [117].

Interestingly, Zychlinsky et al. [118] used *S. flexneri* ∆*ipaB* and ∆*ipaD* mutants as a tool to study adhesion processes and found that these mutants hyperadhere to a variety of host cells, surprisingly, using one pole in contrast with the wild-type that contacted host cells using the long axis. They demonstrated that this hyperadhesion phenotype was due to IcsA, which is localized in a polar manner. Moreover, treatment with the bile salt DOC induced IcsA-dependent adhesion in a T3SS-dependent manner, showing that IcsA becomes adhesive in response to physiological stimuli. 

In addition to IcsA, the multivalent adhesion molecule (MAM) has also been identified as an important adhesin during the earliest stages of infection [119]. MAM is non-redundant with IcsA, so both adhesins may be required during the attachment, and MAM-mediated adherence is a prerequisite for T3SS-mediated invasion of host epithelial and phagocytic cells and necessary for *Shigella* pathogenicity in vivo [119]. 

Mahmoud *et al.* reported that DOC negatively regulates MAM and IcsA in *S. sonnei*, in contrast with what was reported for *S*. *flexneri*. The author speculated that the differential effect of DOC on *S. flexneri* and *S. sonnei* may explain the lower virulence of *S. sonnei*, which indeed requires a higher infectious dose to cause disease [20].

## 4. Immunity to *Shigella*

Observational and challenge studies suggest that immunity to *Shigella* spp. is serotype-specific [120,121,122,123], thus supporting the development of multivalent OAg-based vaccines. In particular, phase 3 studies with the NIH *S. sonnei* glycoconjugate vaccine found a strong association between serum OAg IgG and protective efficacy [124,125,126], and a recent analysis performed by Cohen and colleagues confirmed anti-OAg IgG as potential correlate of protection against shigellosis [127]. After oral administration of live attenuated *Shigella* vaccines and natural infection with wild-type *Shigella*, fecal OAg IgA levels seem instead to correlate with protection, very likely as a result of mucosal immunity in contrast to systemic immunity induced by parenteral vaccines. There are also data suggesting that surface protein antigens, in particular the Ipa proteins, can play a role in protection despite being common to multiple serotypes [128]. Recent data from *Shigella* CHIM studies suggest that anti-IpaB IgG could also be a correlate of protection [129]. It seems that multiple factors can contribute to protection and different immune mechanisms could be required to elicit protective immunity depending on the *Shigella* serotype [130].

In the absence of a correlate of protection, it is important to investigate not only the absolute level of antibodies elicited, but also their functionality. In particular, serum bactericidal assay (SBA) has been proposed for this scope [131,132]. A recent study found a strong association of specific SBA titers against *S. flexneri* 2a in adults with a reduction in disease post-challenge with wild-type organisms [133], supporting the value of this assay to predict vaccine efficacy. Furthermore, bactericidal activity has been detected in sera from individuals naturally exposed to *Shigella* and antibody-dependent complement-mediated killing has been associated with protection against other bacterial pathogens [134]. 

Some studies have also looked at cellular markers of immunity, suggesting that antibody secreting cells, memory B cells, and CD4 + and CD8 + T cells could be additional readouts to be considered [135,136,137].

Since *Shigella* is a human-restricted pathogen, animal infection models currently developed to assess the potential protective efficacy of candidate vaccines, including a murine pulmonary model [138], a cynomolgus monkey *S. dysenteriae* 1 model [139], a guinea pig keratoconjunctivitis model [140], and a guinea pig and piglet oral and intrarectal challenge model [141] do not mirror human infections well. For this reason, testing candidate *Shigella* vaccines in humans seem to be the most valuable way to assess their efficacy. Aligned with this, *Shigella* CHIMs have been established, using either the *S. sonnei* strain 53G or the *S. flexneri* 2a strain 2457T, to provide preliminary estimates of vaccine efficacy in humans [142,143,144]. However, CHIM trials are conducted in adults in high-income countries and the appropriateness to evaluate vaccine efficacy in infants in LMICs based on them is controversial. Furthermore, the conditions of a challenge study are artificial, with higher doses of the challenge agent administered following neutralization of gastric acidity as compared with natural infection [145]. However, CHIMs also provide the opportunity to conduct in-depth immune response characterizations pre- and post-vaccination or pre- and post-infection. Many vaccines based on different technologies are currently in the clinic and moving them to proof of concept phase 2b/phase 3 trials could be important to obtain more data and to better understand key contributors to protective efficacy. These results will also help understand how preclinical immune data can be predictive of human data, supporting the development of current and future vaccines.

## 5. Status of Vaccines in Development

The heterogeneous distribution of *Shigella* serotypes across countries implies that multivalent cross-protective vaccines are required to address the burden of shigellosis. According to GEMS data, a 4-component vaccine consisting of *S. sonnei* and *S. flexneri* 2a, 3a, and 6 OAg could confer direct protection of 64% globally, which could increase to > 85% because of cross-protection against heterologous *S. flexneri* serotypes [32,46]. 

The need for multivalency adds complexity to the development of a vaccine [146] and, at present, there are no licensed vaccines widely available against *Shigella* [147]. Only two *Shigella* vaccines are on the market, but these are limited to Russia (Shigellvak, solution of LPS extracted from *S. sonnei* bacteria) and China (FS, a live attenuated, oral bivalent *S. flexneri* 2a and *S. sonnei* vaccine developed at the Lanzhou Institute of Biological Products). Over the years, potential candidates have included killed, live attenuated, and subunit vaccines.

The earliest vaccines were based on the whole-cell formalin or heat killed approach [148], but these suffered from high levels of reactogenicity. More recently, the Walter Reed Army Institute for Research (WRAIR) developed formalin-inactivated *S. sonnei* (SsWc) [149] and *S. flexneri* 2a (Sf2aWC) [150] monovalent whole-cell vaccine candidates, which were well tolerated in a phase 1 trial, though immune responses were variable. A trivalent version was then developed by PATH and WRAIR [151] containing *S. flexneri* 2a and 3a as well as *S. sonnei*, but these were never tested in clinical trials. A Heat Killed Multi Serotype *Shigella* (HKMS) vaccine is being developed by India’s National Institute of Cholera and Enteric Diseases (NICED) [152] and contains *S. flexneri* 2a, *S. flexneri* 3a, *S. flexneri* 6, *S. sonnei*, *S. dysenteriae* 1, and *S. boydii* 4. This kind of approach has now been largely abandoned.

In the 1960s, Mel and colleagues of the Yugoslav Army developed a live attenuated vaccine that showed protection in military recruits and children aged 2–8 years [153,154]. However, the major challenges of this technology were the need for a high number of doses, relatively short-lived protection, and manufacturing issues, so these vaccines were never licensed. More recently, progresses in whole genome sequencing allowed the development of well-defined live attenuated vaccines with targeted genetic mutations, but balancing between acceptable levels of reactogenicity and sufficient immunogenicity remained a challenge. Among the different vaccine candidates developed, the Center for Vaccine Development (CVD) at the University of Maryland developed a number of *guaBA*-based live attenuated candidates using the *S. flexneri* 2a 2457T strain. In particular, CVD 1208S moved to a phase 2 challenge trial, but after the recruitment of 20 subjects, the study was stopped because of reactogenicity issues [155]. SC602, developed by the Pasteur Institute using *virG* and *iuc* deletions from *S. flexneri* 2a 494 wild-type strain, was mildly reactogenic and immunogenic in US adults and conferred protection against fever, dysentery, and severe symptoms after challenge [156], but it induced a low immune response when tested in adults and children in Bangladesh [157]. WRAIR developed a series of *virG*-based live attenuated vaccines using the wild-type Moseley *S. sonnei* strain (having the plasmid encoding for the OAg relatively stable). The first-generation WRSS1 vaccine resulted as being immunogenic but reactogenic when tested among US and Israeli adults in phase 1 and phase 2 studies [158,159]. WRSS1 was better tolerated in a phase 1 study in Bangladesh, but immune responses were modest, requiring multiple doses and short duration [160]. Moreover, attenuating mutations were introduced to overcome the safety issues resulting in the WRSS2 and WRSS3 candidate vaccines. While both have deleted enterotoxin genes *senA* and *senB*, WRSS3 has *msbB* deleted, resulting in a less acylated and therefore less reactogenic lipid A [161]. Both these vaccines were well tolerated and immunogenic in a phase 1 trial in the US [162] (Table 1).

Several candidates are in preclinical development [175], and recent studies are evaluating *Shigella* genetically modified bacteria, either retaining one OAg repeating unit on the bacterial surface (Δ*wzy*; Truncated *Shigella*, International Vaccine Institute, IVI and PATH) [163] or not displaying LPS-OAg at all (Δ*rfbF*; Eveliqure’s ShigETEC) [164] (Table 1). Both approaches result in increased exposure of well-conserved outer membrane proteins, with the potential to provide broader coverage against multiple *Shigella* serotypes. An innovative vaccine approach, under development by Protein Potential, uses the Ty21a typhoid vaccine displaying *Shigella* LPS; unfortunately, inconsistency in production caused variability in immunogenicity and protection in controlled human infection models (CHIM) studies [165,176].

Parenteral subunit approaches target instead specific *Shigella* antigens. Many of these candidate vaccines are OAg-based. Proof of efficacy was demonstrated 25 years ago with an *S. sonnei* OAg chemically conjugated to a recombinant exotoxin of *Pseudomonas aeruginosa* (*r*EPA) developed by John Robbins at the U.S. National Institutes of Health (NIH) [125,177,178]. Indeed, the vaccine conferred 74% protection in Israeli adults after a single dose [124]. In 2010, protection was also demonstrated in Israeli children aged 3–4 years, but the vaccine failed to protect the younger population [125].

More recently, recombinant glycoconjugates produced in genetically engineered *E. coli* have been proposed, with a bioconjugate against *S. flexneri* 2a, developed by LimmaTech Biologics, proving to be immunogenic in phase 1 clinical trial [179]. In a following CHIM study in US adults, the vaccine did not meet the primary endpoint of protection against all forms of shigellosis but resulted in being protective against more severe shigellosis in a post hoc analysis, and the protection was associated with anti-OAg specific IgG response. Such results allowed further development of a 4-component formulation, S4V-EPA, made of *r*EPA bioconjugates of *S. sonnei* and *S. flexneri* 2a, 3a, and 6, that is currently completing an age-descending dose-finding phase 2 trial in Kenya [166] (Table 1).

The Institute Pasteur has instead developed a well-defined synthetic glycoconjugate vaccine made of synthetically produced *S. flexneri* 2a oligosaccharides chemically linked to tetanus toxoid (TT) carrier protein [177], which was demonstrated to be safe and immunogenic in a phase 1 study in Israeli adults even after a single dose [180]. Sf2a-TT synthetic conjugate is now being tested in an age-descending dose-finding phase 2 trial in Kenya and in a CHIM trial at the CDC [167] (Table 1). 

The traditional glycoconjugate approach has been used by Beijing Zhifei Lvzhu Biopharmaceuticals for the development of a bivalent vaccine, ZF0901, made of *S. sonnei* and *S. flexneri* 2a OAg conjugated to TT. After promising results in phase 1 [181] and phase 2 trials, ZF0901 is currently being tested in a phase 3 study [168] (Table 1).

An interesting alternative subunit vaccine approach has been proposed by WRAIR [169] combining *Shigella* LPS to Ipa proteins. Native Invaplex (InvaplexNAT) contained a complex of IpaB, IpaC, IpaD, and LPS extracted from wild type *S. flexneri* 2a. Intranasal immunization resulted to be well tolerated and immunogenic in phase 1 studies [182,183] but failed to protect in a CHIM trial [184]. Artificial Invaplex (InvaplexAR) was then developed using recombinant IpaB and IpaC produced in *E. coli* and LPS extracted from *S. flexneri* 2a: it was well-tolerated but not consistently immunogenic in a phase 1 study [169]. A new version, the artificial detoxified Invaplex (InvaplexAR-Detox), contains LPS extracted from an *S. flexneri* 2a strain with deleted *msbB* genes. This detoxified form of LPS has allowed its parenteral administration with good safety and immunogenicity results in a recent phase 1 study [169] (Table 1).

GSK Vaccines Institute for Global health (GVGH) has proposed GMMA, outer membrane vesicles (OMVs) from bacteria genetically mutated to increase yields and reduce LPS reactogenicity, for OAg delivery [185]. GMMA resemble the outside of bacteria, where protective antigens, including proteins, are normally found. Moreover, GMMA contain pathogen-associated molecular patterns conferring self-adjuvanticity. The *S. sonnei* monovalent GMMA candidate 1790GAHB has been tested in phase 1 studies in France and the UK [186] and has subsequently been tested in Kenyan adults [187], showing to be well-tolerated and immunogenic and able to boost bactericidal anti-OAg IgG response in naturally exposed or pre-vaccinated subjects [186,188,189]. However, the vaccine failed to elicit protection in a CHIM study on US adults [190]. An improved version of *S. sonnei* GMMA, characterized by higher OAg density, has then been developed and combined with GMMA from *S. flexneri* 1b, 2a, and 3a in a four-component formulation (altSonflex1-2-3) currently being tested in phase 1–2 trials [170] (Table 1).

In addition, the University of Navarra in Spain is developing an acellular *Shigella* vaccine candidate based on OMV encapsulated in nanoparticles, which is currently being tested at the preclinical level [171].

Protein-based subunit vaccine candidates are also under development. They may offer broad protection against all major serotypes but have only been tested in animals so far. Among these, the DB Fusion, developed by PATH, consists of a genetic fusion of the T3SS proteins IpaB and IpaD [172] co-administered intradermally with double mutant Heat Labile Toxin (dmLT) from ETEC with the aim to induce both mucosal and systemic immunity. A 34 kDa OmpA outer membrane protein developed by NICED could provide protection according to preclinical data [173]. IcsP, also named pan-*Shigella* surface protein 1 (PSSP-1), was also suggested as a promising antigen for a broadly protective vaccine against *Shigella* [174].

## 6. Conclusions and Future Perspectives

Vaccination represents an unquestionable solution against infectious diseases, having prevented 700 million cases and more than 150 million deaths during the last century [191]. The Expanded Program on Immunization (EPI) was introduced in 1974 [192], targeting all children throughout the world against different diseases such as diphtheria, tetanus, pertussis, measles, poliomyelitis, tuberculosis, *Haemophilus influenzae* type b, rotavirus, and pneumococcus. In addition, since 2000 the Global Alliance for Vaccines and Immunization (GAVI) has helped to increase childhood immunization coverage in poor countries by supporting the introduction of new vaccines [193]. The latest *Weekly Epidemiological Report* from the WHO Strategic Advisory Group of Experts on Immunization (SAGE) reported the successful introduction of 128 vaccines since 2010 in 86 LMICs [194]. Furthermore, vaccination appears as one of the most promising interventions to fight multidrug-resistant bacterial pathogens effectively [76].

Despite the many years that vaccines have been developed and tested in the clinic against *Shigella*, no licensed vaccine is widely available. *Shigella* is the major bacterial cause of childhood diarrheal death globally, and lack of proper hygiene and sanitation results in repeated diarrheal cases often associated with cognitive impairment and stunting [195,196]. Diagnostic tools and antibiotics are expensive, limited, or not available in LMICs, and according to the current WHO diarrhea treatment guidelines, antibiotics can be given only in case of dysentery, meaning that many children in LMICs with shigellosis do not get antibiotics. Furthermore, as highlighted by the WHO and CDC, the treatment of *Shigella* is increasingly challenging due to the spread of antimicrobial-resistant circulating *Shigella* strains [54,197,198]. All of these factors make the development of a vaccine against *Shigella* a high priority. Because *Shigella* immunity appears to be serotype-specific, the OAg has been proposed as the main vaccine target. The need for multivalency also makes the development of a vaccine against *Shigella* more complex.

Evidence of efficacy was obtained by Mel and colleagues with a live attenuated vaccine, and for many years, efforts have been concentrated on this approach, but live attenuated vaccine candidates suffer from reactogenicity and/or insufficient immunogenicity. Furthermore, these kinds of vaccines require multiple doses and are characterized by a short-lived duration of protection. Later on, efficacy was demonstrated with an *S. sonnei* OAg glycoconjugate vaccine, but the vaccine failed in children below 3 years of age that are the main target for the *Shigella* vaccination.

Recent improvements in the manufacturing of well-defined glycoconjugate vaccines (including bioconjugation and synthetic approach), use of alternative delivery systems (as it is the use of GMMA), and combination of LPS with potentially broad protective protein antigens (e.g., Invaplex) could change the scenario and open the way to the licensure of a *Shigella* vaccine in the near future. Vaccine candidates are now approaching phase 3 clinical trials in infants in endemic countries, and the comparison of different technologies could help identify a correlate of protection. Field efficacy [199] trials should be feasible as the incidence of *Shigella* infection is relatively high in endemic regions, and a quantitative PCR assay has been developed to be more sensitive than traditional culture methods. Discussions with regulatory authorities will be beneficial to maximize the success of these trials and accelerate vaccine implementation. These results will also help to understand how preclinical immune data can be predictive of human data, supporting the development of current and future vaccines.

## Figures and Tables

**Table 1:** Status of vaccine candidates in development against *Shigella*.

Technology	Name	Composition	Developer	Stage	References
Whole-cell Killed Vaccines	SsWc	Formalin-inactivated *S. sonnei*	WRAIR	Discontinued after Ph1	[149]
	Sf2aWC	Formalin-inactivated *S. flexneri* 2a	WRAIR	Discontinued after Ph1	[150]
Live Attenuated Vaccines	FS	*S. flexneri* 2a and *S. sonnei*	Lanzhou Institute of Biological Products	Licensed (limited China)	
	SmD	Streptomycin-dependent *Shigella* strains	Yugoslav Army	Discontinued after Ph3	[154]
	CVD 1208S	*guaBA*-based live attenuated candidates from *S. flexneri* 2a 2457T strain	University of Maryland	Discontinued after Ph2	[155]
	SC602	*virG* and *iuc* deletions from *S. flexneri* 2a 494 wild type strain	Pasteur Institute	Discontinued after Ph2	[157]
	WRSS2	*S. sonnei* Moseley ∆*virG*, *senA* and *senB*	WRAIR	Phase 1	[162]
	WRSS3	*S. sonnei* Moseley ∆*virG*, *senA*, *senB*, and *msBb*			
	Truncated *Shigella*	Attenuated Δ*wzy Shigella flexneri* 2a strain	IVI	Preclinical	[163]
	ShigETEC	*S. flexneri* 2a 2457T ∆*rfbF*, *ipaBC*, and *setBA* expressing fusion protein B subunit of ETEC	EveliQure	Phase 1	[164]
	Ty21a typhoid vaccine expressing *Shigella*	Ty21a typhoid vaccine displaying *Shigella* LPS	Protein Potential	Discontinued after Ph2	[165]
Subunit Vaccines	Shigellvak	*S. sonnei* LPS		Licensed (limited to Russia)	
	*S. sonnei* O-antigen/*r*EPA	*S. sonnei* monovalent O-antigen glycoconjugate	NIH	Discontinued after Ph3	[125]
	S4V-EPA	Quadrivalent *S: flexneri* 2a, 3a, 6 and *S. sonnei* OAg bioconjugate	LimmaTech	Phase 2	[166]
	Sf2a-TT15	*S. flexneri* 2a synthetic OAg conjugate	Institute Pasteur	Phase 2	[167]
	ZF0901	*S. flexneri* 2a and *S. sonnei* OAg conjugate	Beijing Zhifei Lvzhu Biopharmaceuticals	Phase 3	[168]
	Invaplex_AR-_ _DETOX_	LPS from *S. flexneri* 2a 2457T ∆*msBb* and recombinant IpaB and IpaC	WRAIR	Phase 1	[169]
	altSolflex1–2-3	Quadrivalent *S: flexneri* 1b, 2a, 3a, and *S. sonnei* outer membrane vesicles (GMMA)	GVGH (GSK)	Phase 2	[170]
	OMV Sfl2a	*S. flexneri* 2a outer membrane vesicles	Navarra University	Preclinical	[171]
	Ipa DB Fusion	Recombinant protein	PATH	Preclinical	[172]
	34kDa OmpA Sfl2a	Recombinant protein	NICED	Preclinical	[173]
	PSSP-1	Recombinant protein	IVI	Preclinical	[174]

## References

[1] Dadonaite B., Ritchie H., Roser M. (2020). Diarrheal diseases. Our World Data.

[2] Troeger C., Forouzanfar M., Rao P.C., Khalil I., Brown A., Reiner R.C., Fullman N., Thompson R., Abajobir A., Ahmed M. (2017). Estimates of global, regional, and national morbidity, mortality, and aetiologies of diarrhoeal diseases: A systematic analysis for the Global Burden of Disease Study 2015. Lancet Infect. Dis..

[3] World Health Organization (2022). Bacterial Vaccines in Clinical and Preclinical Development 2021: An Overview and Analysis.

[4] Schroeder G.N., Hilbi H. (2008). Molecular pathogenesis of *Shigella* spp.: Controlling host cell signaling, invasion, and death by type III secretion. Clin. Microbiol. Rev..

[5] Shiga K. (1906). Observations on the epidemiology of dysentery in Japan. Philipp. J. Sci..

[6] Liu B., Knirel Y.A., Feng L., Perepelov A.V., Senchenkova S.Y.N., Wang Q., Reeves P.R., Wang L. (2008). Structure and genetics of *Shigella* O antigens. FEMS Microbiol. Rev..

[7] Kotloff K.L., Riddle M.S., Platts-Mills J.A., Pavlinac P., Zaidi A.K.M. (2018). Shigellosis. Lancet.

[8] Rolland K., Lambert-Zechovsky N., Picard B., Denamur E. (1998). *Shigella* and enteroinvasive *Escherichia coli* strains are derived from distinct ancestral strains of *Escherichia coli*. Microbiology.

[9] Fukushima M., Kakinuma K., Kawaguchi R. (2002). Phylogenetic analysis of Salmonella, Shigella, and *Escherichia coli* strains on the basis of the gyrB gene sequence. J. Clin. Microbiol..

[10] Ochman H., Whittam T., Caugant D.A., Selander R.K. (1983). Enzyme polymorphism and genetic population structure in *Escherichia coli* and Shigella. J. Gen. Microbiol..

[11] Lan R., Alles M.C., Donohoe K., Martinez M.B., Reeves P.R. (2004). Molecular evolutionary relationships of enteroinvasive *Escherichia coli* and *Shigella* spp.. Infect. Immun..

[12] Pupo G.M., Lan R., Reeves P.R. (2000). Multiple independent origins of Shigella clones of *Escherichia coli* and convergent evolution of many of their characteristics. Proc. Natl. Acad. Sci. USA.

[13] Jin Q., Yuan Z., Xu J., Wang Y., Shen Y., Lu W., Wang J., Liu H., Yang J., Yang F. (2002). Genome sequence of *Shigella flexneri* 2a: Insights into pathogenicity through comparison with genomes of *Escherichia coli* K12 and O157. Nucleic Acids. Res..

[14] Venkatesan M.M., Goldberg M.B., Rose D.J., Grotbeck E.J., Burland V., Blattner F.R. (2001). Complete DNA sequence and analysis of the large virulence plasmid of *Shigella flexneri*. Infect. Immun..

[15] Yang F., Yang J., Zhang X., Chen L., Jiang Y., Yan Y., Tang X., Wang J., Xiong Z., Dong J. (2005). Genome dynamics and diversity of Shigella species, the etiologic agents of bacillary dysentery. Nucleic Acids. Res..

[16] Maurelli A.T. (2007). Black holes, antivirulence genes, and gene inactivation in the evolution of bacterial pathogens. FEMS Microbiol. Lett..

[17] Jiang Y., Yang F., Zhang X., Yang J., Chen L., Yan Y., Nie H., Xiong Z., Wang J., Dong J. (2005). The complete sequence and analysis of the large virulence plasmid pSS of *Shigella sonnei*. Plasmid.

[18] McVicker G., Tang C.M. (2016). Deletion of toxin-antitoxin systems in the evolution of *Shigella sonnei* as a host-adapted pathogen. Nat. Microbiol..

[19] Killackey S.A., Sorbara M.T., Girardin S.E. (2016). Cellular Aspects of Shigella Pathogenesis: Focus on the Manipulation of Host Cell Processes. Front. Cell. Infect. Microbiol..

[20] DuPont H.L., Levine M.M., Hornick R.B., Formal S.B. (1989). Inoculum Size in Shigellosis and Implications for Expected Mode of Transmission. J. Infect. Dis..

[21] Anderson M., Sansonetti P.J., Marteyn B.S. (2016). Shigella Diversity and Changing Landscape: Insights for the Twenty-First Century. Front. Cell. Infect. Microbiol..

[22] Perdomo O.J.J., Cavaillon J.M., Huerre M., Ohayon H., Gounon P., Sansonetti P.J. (1994). Acute Inflammation Causes Epithelial Invasion and Mucosal Destruction in Experimental Shigellosis. J. Exp. Med..

[23] Phalipon A., Sansonetti P.J. (2007). Shigella’s ways of manipulating the host intestinal innate and adaptive immune system: A tool box for survival?. Immunol. Cell Biol..

[24] Ribet D., Cossart P. (2015). How bacterial pathogens colonize their hosts and invade deeper tissues. Microbes. Infect..

[25] Okuda J., Toyotome T., Kataoka N., Ohno M., Abe H., Shimura Y., Seyedarabi A., Pickersgill R., Sasakawa C. (2005). Shigella effector IpaH9.8 binds to a splicing factor U2AF(35) to modulate host immune responses. Biochem. Biophys. Res. Commun..

[26] Mattock E., Blocker A.J. (2017). How Do the Virulence Factors of Shigella Work Together to Cause Disease?. Front. Cell. Infect. Microbiol..

[27] Ashida H., Mimuro H., Sasakawa C. (2015). Shigella manipulates host immune responses by delivering effector proteins with specific roles. Front. Immunol..

[28] Khalil I.A., Troeger C., Blacker B.F., Rao P.C., Brown A., Atherly D.E., Brewer T.G., Engmann C.M., Houpt E.R., Kang G. (2018). Morbidity and mortality due to shigella and enterotoxigenic *Escherichia coli* diarrhoea: The Global Burden of Disease Study 1990–2016. Lancet Infect. Dis..

[29] Liu J., Platts-Mills J.A., Juma J., Kabir F., Nkeze J., Okoi C., Operario D.J., Uddin J., Ahmed S., Alonso P.L. (2016). Use of quantitative molecular diagnostic methods to identify causes of diarrhoea in children: A reanalysis of the GEMS case-control study. Lancet.

[30] Marder E.P., Cieslak P.R., Cronquist A.B., Dunn J., Lathrop S., Rabatsky-Ehr T., Ryan P., Smith K., Tobin-D’Angelo M., Vugia D.J. (2017). Incidence and Trends of Infections with Pathogens Transmitted Commonly Through Food and the Effect of Increasing Use of Culture-Independent Diagnostic Tests on Surveillance—Foodborne Diseases Active Surveillance Network, 10 U.S. Sites, 2013–2016. Morb. Mortal. Wkly. Rep..

[31] Kotloff K.L., Nataro J.P., Blackwelder W.C., Nasrin D., Farag T.H., Panchalingam S., Wu Y., Sow S.O., Sur D., Breiman R.F. (2013). Burden and aetiology of diarrhoeal disease in infants and young children in developing countries (the Global Enteric Multicenter Study, GEMS): A prospective, case-control study. Lancet.

[32] Livio S., Strockbine N.A., Panchalingam S., Tennant S.M., Barry E.M., Marohn M.E., Antonio M., Hossain A., Mandomando I., Ochieng J.B. (2014). Shigella Isolates from the Global Enteric Multicenter Study Inform Vaccine Development. Clin. Infect. Dis..

[33] Kahsay A.G., Muthupandian S. (2016). A review on Sero diversity and antimicrobial resistance patterns of Shigella species in Africa, Asia and South America, 2001–2014. BMC Res Notes.

[34] Puzari M., Sharma M., Chetia P. (2018). Emergence of antibiotic resistant Shigella species: A matter of concern. J. Infect. Public Health.

[35] Qu F., Bao C., Chen S., Cui E., Guo T., Wang H., Zhang J., Wang H., Tang Y.W., Mao Y. (2012). Genotypes and antimicrobial profiles of *Shigella sonnei* isolates from diarrheal patients circulating in Beijing between 2002 and 2007. Diagn. Microbiol. Infect. Dis..

[36] Thompson C.N., Duy P.T., Baker S. (2015). The Rising Dominance of *Shigella sonnei*: An Intercontinental Shift in the Etiology of Bacillary Dysentery. PLoS Negl. Trop. Dis..

[37] Vinh H., Nhu N.T., Nga T.V., Duy P.T., Campbell J.I., Hoang N.V., Boni M.F., My P.V., Parry C., Nga T.T. (2009). A changing picture of shigellosis in southern Vietnam: Shifting species dominance, antimicrobial susceptibility and clinical presentation. BMC Infect. Dis..

[38] Bangtrakulnonth A., Vieira A.R., Lo Fo Wong D.M.A., Pornreongwong S., Pulsrikarn S., Sawanpanyalert P., Hendriksen R.S., Aarestrup F.M. (2008). Shigella from Humans in Thailand During 1993 to 2006: Spatial-Time Trends in Species and Serotype Distribution. Foodborne Pathog. Dis..

[39] Ud-Din A.I., Wahid S.U., Latif H.A., Shahnaij M., Akter M., Azmi I.J., Hasan T.N., Ahmed D., Hossain M.A., Faruque A.S. (2013). Changing trends in the prevalence of Shigella species: Emergence of multi-drug resistant *Shigella sonnei* biotype g in Bangladesh. PLoS ONE.

[40] Zarei M., Ghahfarokhi M.E., Fazlara A., Bahrami S. (2019). Effect of the bacterial growth phase and coculture conditions on the interaction of *Acanthamoeba castellanii* with *Shigella dysenteriae*, *Shigella flexneri*, and *Shigella sonnei*. J. Basic Microbiol..

[41] Niyogi S. (2005). Shigellosis. J. Microbiol. Immunol. Infect..

[42] Rolfo F., Marin G.H., Silberman M., Pattin J., Giugnio S., Gatti B., Bettiol M., Rigoni A. (2012). Epidemiological study of shigellosis in an urban area of Argentina. J. Infect. Dis. Dev. Ctries..

[43] Fernandez-Prada C.M., Venkatesan M.M., Franco A.A., Lanata C.F., Sack R.B., Hartman A.B., Spira W. (2004). Molecular epidemiology of *Shigella flexneri* in a diarrhoea-endemic area of Lima, Peru. Epidemiol. Infect..

[44] Taneja N., Mewara A. (2016). Shigellosis: Epidemiology in India. Indian J. Med. Res..

[45] Muthuirulandi Sethuvel D.P., Devanga Ragupathi N.K., Anandan S., Veeraraghavan B. (2017). Update on: Shigella new serogroups/serotypes and their antimicrobial resistance. Lett. Appl. Microbiol..

[46] Mani S., Wierzba T., Walker R.I. (2016). Status of vaccine research and development for Shigella. Vaccine.

[47] Bardhan P., Faruque A.S., Naheed A., Sack D.A. (2010). Decrease in shigellosis-related deaths without *Shigella* spp.-specific interventions, Asia. Emerg. Infect. Dis..

[48] Gu B., Cao Y., Pan S., Zhuang L., Yu R., Peng Z., Qian H., Wei Y., Zhao L., Liu G. (2012). Comparison of the prevalence and changing resistance to nalidixic acid and ciprofloxacin of Shigella between Europe-America and Asia-Africa from 1998 to 2009. Int. J. Antimicrob. Agents.

[49] Shah N., DuPont H.L., Ramsey D.J. (2009). Global Etiology of Travelers’ Diarrhea: Systematic Review from 1973 to the Present *Am*. J. Trop. Med. Hyg..

[50] Bowen A., Hurd J., Hoover C., Khachadourian Y., Traphagen E., Harvey E., Libby T., Ehlers S., Ongpin M., Norton J.C. (2015). Importation and Domestic Transmission of *Shigella sonnei* Resistant to Ciprofloxacin—United States, May 2014–February 2015. Morb. Mortal. Wkly. Rep..

[51] Olson S., Hall A., Riddle M.S., Porter C.K. (2019). Travelers’ diarrhea: Update on the incidence, etiology and risk in military and similar populations—1990–2005 versus 2005–2015, does a decade make a difference?. Trop. Dis. Travel Med. Vaccines.

[52] Ouyang-Latimer J., Jafri S., VanTassel A., Jiang Z.D., Gurleen K., Rodriguez S., Nandy R.K., Ramamurthy T., Chatterjee S., McKenzie R. (2011). In vitro antimicrobial susceptibility of bacterial enteropathogens isolated from international travelers to Mexico, Guatemala, and India from 2006 to 2008. Antimicrob. Agents Chemother..

[53] Tribble D.R. (2017). Resistant pathogens as causes of traveller’s diarrhea globally and impact(s) on treatment failure and recommendations. J. Travel Med..

[54] WHO Publishes List of Bacteria for Which New Antibiotics Are Urgently Needed. https://www.who.int/news/item/27-02-2017-who-publishes-list-of-bacteria-for-which-new-antibiotics-are-urgently-needed.

[55] Taitt C.R., Leski T.A., Prouty M.G., Ford G.W., Heang V., House B.L., Levin S.Y., Curry J.A., Mansour A., Mohammady H.E. (2020). Tracking Antimicrobial Resistance Determinants in Diarrheal Pathogens: A Cross-Institutional Pilot Study. Int. J. Mol. Sci..

[56] Qiu S., Wang Y., Xu X., Li P., Hao R., Yang C., Liu N., Li Z., Wang Z., Wang J. (2013). Multidrug-resistant atypical variants of *Shigella flexneri* in China. Emerg. Infect. Dis..

[57] Marshall E.K., Bratton A.C., White H.J., Litchfield J.T. (1940). Sulfanilylguanidine: A chemotherapeutic agent for intestinal infections. Bull. Johns Hopkins Hosp..

[58] Neter E. (1948). The genus Shigella and shigellosis. Am. J. Dis. Child.

[59] Ross S., Burke F.G., Rice E.C., Washington J.A., Stevens S. (1950). Chloramphenicol (Chloromycetin) therapy in *Shigella enteritis*. JAMA.

[60] Klontz K.C., Singh N. (2015). Treatment of drug-resistant Shigella infections. Expert Rev. Anti-Infect. Ther..

[61] Chung The H., Boinett C., Pham Thanh D., Jenkins C., Weill F.X., Howden B.P., Valcanis M., De Lappe N., Cormican M., Wangchuk S. (2019). Dissecting the molecular evolution of fluoroquinolone-resistant *Shigella sonnei*. Nat. Commun..

[62] Mahbubur R., Shoma S., Rashid H., Arifeen S.E., Baqui A.H., Siddique A.K., Nair G.B., Sack D.A. (2007). Increasing spectrum in antimicrobial resistance of Shigella isolates in Bangladesh: Resistance to azithromycin and ceftriaxone and decreased susceptibility to ciprofloxacin. Health Popul. Nutr..

[63] Salimiyan Rizi K., Farsiani H., Sasan M.S. (2020). High rate of resistance to ceftriaxone and azithromycin among *Shigella* spp. isolates at three children’s referral hospitals in Northeast Iran. J. Infect. Chemother..

[64] Raja S.B., Murali M.R., Devaraj S.N. (2008). Differential expression of ompC and ompF in multidrug-resistant *Shigella dysenteriae* and *Shigella flexneri* by aqueous extract of Aegle marmelos, altering its susceptibility toward beta-lactam antibiotics. Diagn. Microbiol. Infect. Dis..

[65] Bhattacharya D., Bhattacharya H., Thamizhmani R., Sayi D.S., Reesu R., Anwesh M., Kartick C., Bharadwaj A.P., Singhania M., Sugunan A.P. (2014). Shigellosis in Bay of Bengal Islands, India: Clinical and seasonal patterns, surveillance of antibiotic susceptibility patterns, and molecular characterization of multidrug-resistant Shigella strains isolated during a 6-year period from 2006 to 2011. Eur. J. Clin. Microbiol. Infect. Dis. Off. Publ. Eur. Soc. Clin. Microbiol..

[66] Shahsavan S., Owlia P., Lari A.R., Bakhshi B., Nobakht M. (2017). Investigation of efflux-mediated tetracycline resistance in Shigella isolates using the inhibitor and real time polymerase chain reaction method. I. Iran. J. Pathol..

[67] Traa B.S., Walker C.L., Munos M., Black R.E. (2010). Antibiotics for the treatment of dysentery in children. Int. J. Epidemiol..

[68] Kar A.K., Ghosh A.S., Chauhan K., Ahamed J., Basu J., Chakrabarti P., Kundu M. (1997). Involvement of a 43-kilodalton outer membrane protein in beta-lactam resistance of *Shigella dysenteriae*. Antimicrob. Agents. Chemother..

[69] Tran E.N., Papadopoulos M., Morona R. (2014). Relationship between O-antigen chain length and resistance to colicin E2 in *Shigella flexneri*. Microbiology.

[70] Ghosh A.S., Kar A.K., Kundu M. (1999). Impaired imipenem uptake associated with alterations in outer membrane proteins and lipopolysaccharides in imipenem-resistant *Shigella dysenteriae J*. Antimicrob. Chemother..

[71] Williams P.C.M., Berkley J.A. (2018). Guidelines for the treatment of dysentery (shigellosis): A systematic review of the evidence. Paediatr. Int. Child Health.

[72] Ranjbar R., Farahani A. (2019). Shigella: Antibiotic-Resistance Mechanisms And New Horizons For Treatment. Infect. Drug Resist..

[73] Filho-Lima J.V.M., Vieira E.C., Nicoli J.R. (2000). Antagonistic effect of Lactobacillus acidophilus, Saccharomyces boulardii and *Escherichia coli* combinations against experimental infections with *Shigella flexneri* and *Salmonella enteritidis* subsp. typhimurium in gnotobiotic mice. J. Appl. Microbiol..

[74] Saqib S., Munis M.F.H., Zaman W., Ullah F., Shah S.N., Ayaz A., Farooq M., Bahadur S. (2019). Synthesis, characterization and use of iron oxide nano particles for antibacterial activity. Microsc. Res. Tech..

[75] Jamal M., Chaudhry W.N., Hussain T., Das C.R., Andleeb S. (2015). Characterization of new myoviridae bacteriophage WZ1 against multi-drug resistant (MDR) *Shigella dysenteriae*. J. Basic Microbiol..

[76] Micoli F., Bagnoli F., Rappuoli R., Serruto D. (2021). The role of vaccines in combatting antimicrobial resistance. Nat. Rev. Microbiol..

[77] Knirel Y.A., Kondakova A.N., Vinogradov E., Lindner B., Perepelov A.V., Shashkov A.S. (2011). Lipopolysaccharide core structures and their correlation with genetic groupings of Shigella strains. A novel core variant in *Shigella boydii* type 16. Glycobiology.

[78] Knirel Y.A., Lan R., Senchenkova S.N., Wang J., Shashkov A.S., Wang Y., Perepelov A.V., Xiong Y., Xu J., Sun Q. (2013). O-antigen structure of *Shigella flexneri* serotype Yv and effect of the lpt-O gene variation on phosphoethanolamine modification of *S. flexneri* O-antigens. Glycobiology.

[79] Perepelov A.V., Shekht M.E., Liu B., Shevelev S.D., Ledov V.A., Senchenkova S.N., L’vov V.L., Shashkov A.S., Feng L., Aparin P.G. (2012). *Shigella flexneri* O-antigens revisited: Final elucidation of the O-acetylation profiles and a survey of the O-antigen structure diversity. FEMS Immunol. Med. Microbiol..

[80] Kubler-Kielb J., Vinogradov E., Mocca C., Pozsgay V., Coxon B., Robbins J.B., Schneerson R. (2010). Immunochemical studies of *Shigella flexneri* 2a and 6, and *Shigella dysenteriae* type 1 O-specific polysaccharide-core fragments and their protein conjugates as vaccine candidates. Carbohydr. Res..

[81] Robbins J.B., Kubler-Kielb J., Vinogradov E., Mocca C., Pozsgay V., Shiloach J., Schneerson R. (2009). Synthesis, characterization, and immunogenicity in mice of *Shigella sonnei* O-specific oligosaccharide-core-protein conjugates. Proc. Natl. Acad. Sci. USA.

[82] Islam S.T., Lam J.S. (2014). Synthesis of bacterial polysaccharides via the Wzx/Wzy-dependent pathway. Can. J. Microbiol..

[83] Caboni M., Pedron T., Rossi O., Goulding D., Pickard D., Citiulo F., MacLennan C.A., Dougan G., Thomson N.R., Saul A. (2015). An O antigen capsule modulates bacterial pathogenesis in *Shigella sonnei*. PLoS Pathog..

[84] Raso M.M., Gasperini G., Alfini R., Schiavo F., Aruta M.G., Carducci M., Forgione M.C., Martini S., Cescutti P., Necchi F. (2020). GMMA and Glycoconjugate Approaches Compared in Mice for the Development of a Vaccine against *Shigella flexneri* Serotype 6. Vaccines.

[85] Gasperini G., Raso M.M., Arato V., Aruta M.G., Cescutti P., Necchi F., Micoli F. (2021). Effect of O-Antigen Chain Length Regulation on the Immunogenicity of Shigella and Salmonella Generalized Modules for Membrane Antigens (GMMA). Int. J. Mol. Sci..

[86] Knirel Y.A., Sun Q., Senchenkova S.N., Perepelov A.V., Shashkov A.S., Xu J. (2015). O-antigen modifications providing antigenic diversity of *Shigella flexneri* and underlying genetic mechanisms. Biochemistry.

[87] West N.P., Sansonetti P., Mounier J., Exley R.M., Parsot C., Guadagnini S., Prevost M.C., Prochnicka-Chalufour A., Delepierre M., Tanguy M. (2005). Optimization of virulence functions through glucosylation of Shigella LPS. Science.

[88] Sansonetti P.J., Kopecko D.J., Formal S.B. (1982). Involvement of a plasmid in the invasive ability of *Shigella flexneri*. Infect. Immun..

[89] Ogawa M., Sasakawa C. (2006). Intracellular survival of Shigella. Cell. Microbiol..

[90] Parsot C. (2005). *Shigella* spp. and enteroinvasive *Escherichia coli* pathogenicity factors. FEMS Microbiol. Lett..

[91] Muthuramalingam M., Whittier S.K., Picking W.L., Picking W.D. (2021). The Shigella Type III Secretion System: An Overview from Top to Bottom. Microorganisms.

[92] Zenk S.F., Stabat D., Hodgkinson J.L., Veenendaal A.K.J., Johnson S., Blocker A.J. (2007). Identification of minor inner-membrane components of the Shigella type III secretion system ‘needle complex’. Microbiology.

[93] Blocker A., Gounon P., Larquet E., Niebuhr K., Cabiaux V., Parsot C., Sansonetti P. (1999). The Tripartite Type III Secreton of *Shigella flexneri* Inserts IpaB and IpaC into Host Membranes. J. Cell Biol..

[94] Hayward R.D., Cain R.J., McGhie E.J., Phillips N., Garner M.J., Koronakis V. (2005). Cholesterol binding by the bacterial type III translocon is essential for virulence effector delivery into mammalian cells. Mol. Microbiol..

[95] Epler C.R., Dickenson N.E., Olive A.J., Picking W.L., Picking W.D. (2009). Liposomes recruit IpaC to the *Shigella flexneri* type III secretion apparatus needle as a final step in secretion induction. Infect. Immun..

[96] Verasdonck J., Shen D.K., Treadgold A., Arthur C., Böckmann A., Meier B.H., Blocker A.J. (2015). Reassessment of MxiH subunit orientation and fold within native Shigella T3SS needles using surface labelling and solid-state NMR. J. Struct. Biol..

[97] McDowell M.A., Marcoux J., McVicker G., Johnson S., Fong Y.H., Stevens R., Bowman L.A., Degiacomi M.T., Yan J., Wise A. (2016). Characterisation of Shigella Spa33 and Thermotoga FliM/N reveals a new model for C-ring assembly in T3SS. Mol. Microbiol..

[98] Hu B., Morado D.R., Margolin W., Rohde J.R., Arizmendi O., Picking W.L., Picking W.D., Liu J. (2015). Visualization of the type III secretion sorting platform of *Shigella flexneri*. Proc. Natl. Acad. Sci. USA.

[99] Gao X., Mu Z., Yu X., Qin B., Wojdyla J., Wang M., Cui S. (2018). Structural Insight Into Conformational Changes Induced by ATP Binding in a Type III Secretion-Associated ATPase From *Shigella flexneri*. Front. Microbiol..

[100] Cherradi Y., Hachani A., Allaoui A. (2014). Spa13 of *Shigella flexneri* has a dual role: Chaperone escort and export gate-activator switch of the type III secretion system. Microbiology.

[101] Francis M.S., Wolf-Watz H., Forsberg A. (2002). Regulation of type III secretion systems. Curr. Opin. Microbiol..

[102] McKenna J.A., Karney M.M.A., Chan D.K., Weatherspoon-Griffin N., Becerra Larios B., Pilonieta M.C., Munson G.P., Wing H.J. (2022). The AraC/XylS Protein MxiE and Its Coregulator IpgC Control a Negative Feedback Loop in the Transcriptional Cascade That Regulates Type III Secretion in *Shigella flexneri*. J. Bacteriol..

[103] Perwaiz S., Tuchweber B., Mignault D., Gilat T., Yousef I.M. (2001). Determination of bile acids in biological fluids by liquid chromatography-electrospray tandem mass spectrometry. J. Lipid Res..

[104] Faherty C.S., Redman J.C., Rasko D.A., Barry E.M., Nataro J.P. (2012). *Shigella flexneri* effectors OspE1 and OspE2 mediate induced adherence to the colonic epithelium following bile salts exposure. Mol. Microbiol..

[105] Olive A.J., Kenjale R., Espina M., Moore D.S., Picking W.L., Picking W.D. (2007). Bile salts stimulate recruitment of IpaB to the *Shigella flexneri* surface, where it colocalizes with IpaD at the tip of the type III secretion needle. Infect. Immun..

[106] Dickenson N.E., Zhang L., Epler C.R., Adam P.R., Picking W.L., Picking W.D. (2011). Conformational changes in IpaD from *Shigella flexneri* upon binding bile salts provide insight into the second step of type III secretion. Biochemistry.

[107] Dickenson N.E., Arizmendi O., Patil M.K., Toth IV R.T., Middaugh C.R., Picking W.D., Picking W.L. (2013). N-terminus of IpaB provides a potential anchor to the Shigella type III secretion system tip complex protein IpaD. Biochemistry.

[108] Kline K.A., Falker S., Dahlberg S., Normark S., Henriques-Normark B. (2009). Bacterial adhesins in host-microbe interactions. Cell Host Microbe.

[109] Goldberg M.B., Theriot J.A. (1995). *Shigella flexneri* surface protein IcsA is sufficient to direct actin-based motility. Proc. Natl. Acad. Sci. USA.

[110] Purdy G.E., Hong M., Payne S.M. (2002). *Shigella flexneri* DegP facilitates IcsA surface expression and is required for efficient intercellular spread. Infect. Immun..

[111] Purdy G.E., Fisher C.R., Payne S.M. (2007). IcsA surface presentation in *Shigella flexneri* requires the periplasmic chaperones DegP, Skp, and SurA. J. Bacteriol..

[112] Egile C., d’Hauteville H., Parsot C., Sansonetti P.J. (1997). SopA, the outer membrane protease responsible for polar localization of IcsA in *Shigella flexneri*. Mol. Microbiol..

[113] Ambrosi C., Pompili M., Scribano D., Zagaglia C., Ripa S., Nicoletti M. (2012). Outer membrane protein A (OmpA): A new player in *Shigella flexneri* protrusion formation and inter-cellular spreading. PLoS ONE.

[114] Janakiraman A., Fixen K.R., Gray A.N., Niki H., Goldberg M.B. (2009). A genome-scale proteomic screen identifies a role for DnaK in chaperoning of polar autotransporters in *Shigella*. J. Bacteriol..

[115] Gray A.N., Li Z., Henderson-Frost J., Goldberg M.B. (2014). Biogenesis of YidC cytoplasmic membrane substrates is required for positioning of autotransporter IcsA at future poles. J. Bacteriol..

[116] Santapaola D., Del Chierico F., Petrucca A., Uzzau S., Casalino M., Colonna B., Sessa R., Berlutti F., Nicoletti M. (2006). Apyrase, the product of the virulence plasmid-encoded phoN2 (apy) gene of *Shigella flexneri*, is necessary for proper unipolar IcsA localization and for efficient intercellular spread. J. Bacteriol..

[117] Africa L.A., Murphy E.R., Egan N.R., Wigley A.F., Wing H.J. (2011). The iron-responsive Fur/RyhB regulatory cascade modulates the Shigella outer membrane protease IcsP. Infect. Immun..

[118] Brotcke Zumsteg A., Goosmann C., Brinkmann V., Morona R., Zychlinsky A. (2014). IcsA is a *Shigella flexneri* adhesin regulated by the type III secretion system and required for pathogenesis. Cell Host Microbe.

[119] Mahmoud R.Y., Stones D.H., Li W., Emara M., El-Domany R.A., Wang D., Wang Y., Krachler A.M., Yu J. (2016). The Multivalent Adhesion Molecule SSO1327 plays a key role in *Shigella sonnei* pathogenesis. Mol. Microbiol..

[120] Cohen D., Meron-Sudai S., Bialik A., Asato V., Ashkenazi S. (2022). Detoxified O-Specific Polysaccharide (O-SP)-Protein Conjugates: Emerging Approach in the Shigella Vaccine Development Scene. Vaccines.

[121] Formal S.B., Oaks E.V., Olsen R.E., Wingfield-Eggleston M., Snoy P.J., Cogan J.P. (1991). Effect of prior infection with virulent *Shigella flexneri* 2a on the resistance of monkeys to subsequent infection with *Shigella sonnei*. J. Infect. Dis..

[122] DuPont H.L., Hornick R.B., Snyder M.J., Libonati J.P., Formal S.B., Gangarosa E.J. (1972). Immunity in Shigellosis. II. Protection Induced by Oral Live Vaccine or Primary Infection. J. Infect. Dis..

[123] Cohen D., Bassal R., Goren S., Rouach T., Taran D., Schemberg B., Peled N., Keness Y., Ken-Dror S., Vasilev V. (2014). Recent trends in the epidemiology of shigellosis in Israel. Epidemiol. Infect..

[124] Cohen D., Ashkenazi S., Green M.S., Gdalevich M., Robin G., Slepon R., Yavzori M., Orr N., Block C., Ashkenazi I. (1997). Double-blind vaccine-controlled randomised efficacy trial of an investigational *Shigella sonnei* conjugate vaccine in young adults. Lancet.

[125] Passwell J.H., Ashkenazi S., Banet-Levi Y., Ramon-Saraf R., Farzam N., Lerner-Geva L., Even-Nir H., Yerushalmi B., Chu C., Shiloach J. (2010). Age-related efficacy of Shigella O-specific polysaccharide conjugates in 1–4-year-old Israeli children. Vaccine.

[126] Ménard R., Prévost M.C., Gounon P., Sansonetti P., Dehio C. (1996). The secreted Ipa complex of *Shigella flexneri* promotes entry into mammalian cells. Proc. Natl. Acad. Sci. USA.

[127] Cohen D., Meron-Sudai S., Bialik A., Asato V., Goren S., Ariel-Cohen O., Reizis A., Hochberg A., Ashkenazi S. (2019). Serum IgG antibodies to Shigella lipopolysaccharide antigens—A correlate of protection against shigellosis. Hum. Vaccin. Immunother..

[128] Cohen D., Ashkenazi S., Schneerson R., Farzam N., Bialik A., Meron-Sudai S., Asato V., Goren S., Baran T.Z., Muhsen K. (2022). Threshold Protective Levels of Serum IgG to *Shigella* Lipopolysaccharide: Re-Analysis of *Shigella* Vaccine Trials Data. Clin. Microbiol. Infect..

[129] Ndungo E., Randall A., Hazen T.H., Kania D.A., Trappl-Kimmons K., Liang X., Barry E.M., Kotloff K.L., Chakraborty S., Mani S. (2018). A Novel Shigella Proteome Microarray Discriminates Targets of Human Antibody Reactivity following Oral Vaccination and Experimental Challenge. Msphere.

[130] Clarkson K.A., Porter C.K., Talaat K.R., Frenck R.W., Alaimo C., Martin P., Bourgeois A.L., Kaminski R.W. (2021). Shigella-Specific Immune Profiles Induced after Parenteral Immunization or Oral Challenge with Either *Shigella flexneri* 2a or *Shigella sonnei*. Msphere.

[131] Nahm M.H., Yu J., Weerts H.P., Wenzel H., Tamilselvi C.S., Chandrasekaran L., Pasetti M.F., Mani S., Kaminski R.W. (2018). Development, Interlaboratory Evaluations, and Application of a Simple, High-Throughput *Shigella* Serum Bactericidal Assay. Msphere.

[132] Rossi O., Molesti E., Saul A., Giannelli C., Micoli F., Necchi F. (2020). Intra-Laboratory Evaluation of Luminescence Based High-Throughput Serum Bactericidal Assay (L-SBA) to Determine Bactericidal Activity of Human Sera against *Shigella*. High-Throughput.

[133] Shimanovich A.A., Buskirk A.D., Heine S.J., Blackwelder W.C., Wahid R., Kotloff K.L., Pasetti M.F. (2017). Functional and Antigen-Specific Serum Antibody Levels as Correlates of Protection against Shigellosis in a Controlled Human Challenge Study. Clin. Vaccine Immunol..

[134] Goldschneider I., Gotschlich E.C., Artenstein M.S. (1969). Human immunity to the meningococcus. I. The role of humoral antibodies. J. Exp. Med..

[135] Van de Verg L., Herrington D.A., Murphy J.R., Wasserman S.S., Formal S.B., Levine M.M. (1990). Specific immunoglobulin A-secreting cells in peripheral blood of humans following oral immunization with a bivalent Salmonella typhi-*Shigella sonnei* vaccine or infection by pathogenic *S. sonnei*. Infect. Immun..

[136] Simon J.K., Maciel M., Weld E.D., Wahid R., Pasetti M.F., Picking W.L., Kotloff K.L., Levine M.M., Sztein M.B. (2011). Antigen-specific IgA B memory cell responses to Shigella antigens elicited in volunteers immunized with live attenuated *Shigella flexneri* 2a oral vaccine candidates. Clin. Immunol..

[137] Toapanta F.R., Bernal P.J., Kotloff K.L., Levine M.M., Sztein M.B. (2018). T cell mediated immunity induced by the live-attenuated *Shigella flexneri* 2a vaccine candidate CVD 1208S in humans. J. Transl. Med..

[138] Mallett C.P., VanDeVerg L., Collins H.H., Hale T.L. (1993). Evaluation of Shigella vaccine safety and efficacy in an intranasally challenged mouse model. Vaccine.

[139] Shipley S.T., Panda A., Khan A.Q., Kriel E.H., Maciel M., Livio S., Nataro J.P., Levine M.M., Sztein M.B., DeTolla L.J. (2010). A challenge model for *Shigella dysenteriae* 1 in cynomolgus monkeys (*Macaca fascicularis*). Comp. Med..

[140] Shim D.H., Suzuki T., Chang S.Y., Park S.M., Sansonetti P.J., Sasakawa C., Kweon M.N. (2007). New animal model of shigellosis in the Guinea pig: Its usefulness for protective efficacy studies. J. Immunol..

[141] Barman S., Saha D.R., Ramamurthy T., Koley H. (2011). Development of a new guinea-pig model of shigellosis. FEMS Immunol. Med. Microbiol..

[142] Black R.E., Levine M.M., Clements M.L., Losonsky G., Herrington D., Berman S., Formal S.B. (1987). Prevention of shigellosis by a Salmonella typhi-*Shigella sonnei* bivalent vaccine. J. Infect. Dis..

[143] Frenck R.W., Dickey M., Suvarnapunya A.E., Chandrasekaran L., Kaminski R.W., Clarkson K.A., McNeal M., Lynen A., Parker S., Hoeper A. (2020). Establishment of a Controlled Human Infection Model with a Lyophilized Strain of *Shigella sonnei* 53G. Msphere.

[144] Talaat K.R., Alaimo C., Martin P., Bourgeois A.L., Dreyer A.M., Kaminski R.W., Porter C.K., Chakraborty S., Clarkson K.A., Brubaker J. (2021). Human challenge study with a Shigella bioconjugate vaccine: Analyses of clinical efficacy and correlate of protection. EBioMedicine.

[145] Giersing B.K., Porter C.K., Kotloff K., Neels P., Cravioto A., MacLennan C.A. (2019). How can controlled human infection models accelerate clinical development and policy pathways for vaccines against Shigella?. Vaccine.

[146] Levine M.M., Kotloff K.L., Barry E.M., Pasetti M.F., Sztein M.B. (2007). Clinical trials of Shigella vaccines: Two steps forward and one step back on a long, hard road. Nat. Rev. Microbiol..

[147] MacLennan C.A., Grow S., Ma L.F., Steele A.D. (2022). The Shigella Vaccines Pipeline. Vaccines.

[148] Herrera C.M., Schmitt J.S., Chowdhry E.I., Riddle M.S. (2022). From Kiyoshi Shiga to Present-Day Shigella Vaccines: A Historical Narrative Review. Vaccines.

[149] McKenzie R., Walker R.I., Nabors G.S., Van De Verg L.L., Carpenter C., Gomes G., Forbes E., Tian J.H., Yang H.H., Pace J.L. (2006). Safety and immunogenicity of an oral, inactivated, whole-cell vaccine for *Shigella sonnei*: Preclinical studies and a Phase I trial. Vaccine.

[150] Chakraborty S., Harro C., DeNearing B., Bream J., Bauers N., Dally L., Flores J., Van de Verg L., Sack D.A., Walker R. (2016). Evaluation of the Safety, Tolerability, and Immunogenicity of an Oral, Inactivated Whole-Cell *Shigella flexneri* 2a Vaccine in Healthy Adult Subjects. Clin. Vaccine Immunol..

[151] Kaminski R.W., Wu M., Turbyfill K.R., Clarkson K., Tai B., Bourgeois A.L., Van De Verg L.L., Walker R.I., Oaks E.V. (2014). Development and preclinical evaluation of a trivalent, formalin-inactivated Shigella whole-cell vaccine. Clin. Vaccine. Immunol..

[152] Nag D., Sinha R., Mitra S., Barman S., Takeda Y., Shinoda S., Chakrabarti M.K., Koley H. (2015). Heat killed multi-serotype Shigella immunogens induced humoral immunity and protection against heterologous challenge in rabbit model. Immunobiology.

[153] Mel D.M., Arsić B.L., Nikolić B.D., Radovanić M.L. (1968). Studies on vaccination against bacillary dysentery. 4. Oral immunization with live monotypic and combined vaccines. Bull. World Health Organ..

[154] Mel D., Gangarosa E.J., Radovanovic M.L., Arsic B.L., Litvinjenko S. (1971). Studies on vaccination against bacillary dysentery. 6. Protection of children by oral immunization with streptomycin-dependent Shigella strains. Bull. World Health Organ..

[155] Kotloff K. Safety and Efficacy Study of CVD 1208S, a Live, Attenuated Oral Vaccine to Prevent Shigella Infection: Phase IIa. https://clinicaltrials.gov/ct2/show/record/NCT00866476?cond=shigella&draw=2&rank=4&view=record.

[156] Coster T.S., Hoge C.W., VanDeVerg L.L., Hartman A.B., Oaks E.V., Venkatesan M.M., Cohen D., Robin G., Fontaine-Thompson A., Sansonetti P.J. (1999). Vaccination against Shigellosis with Attenuated *Shigella flexneri* 2a Strain SC602. Infect. Immun..

[157] Rahman K.M., Arifeen S.E., Zaman K., Rahman M., Raqib R., Yunus M., Begum N., Islam M.S., Sohel B.M., Rahman M. (2011). Safety, dose, immunogenicity, and transmissibility of an oral live attenuated *Shigella flexneri* 2a vaccine candidate (SC602) among healthy adults and school children in Matlab, Bangladesh. Vaccine.

[158] Kotloff K.L., Taylor D.N., Sztein M.B., Wasserman S.S., Losonsky G.A., Nataro J.P., Venkatesan M., Hartman A., Picking W.D., Katz D.E. (2002). Phase I Evaluation of Δ*virG Shigella sonnei* Live, Attenuated, Oral Vaccine Strain WRSS1 in Healthy Adults. Infect. Immun..

[159] Orr N., Katz D.E., Atsmon J., Radu P., Yavzori M., Halperin T., Sela T., Kayouf R., Klein Z., Ambar R. (2005). Community-Based Safety, Immunogenicity, and Transmissibility Study of the *Shigella sonnei* WRSS1 Vaccine in Israeli Volunteers. Infect. Immun..

[160] Raqib R., Sarker P., Zaman K., Alam N.H., Wierzba T.F., Maier N., Talukder K., Baqui A.H., Suvarnapunya A.E., Qadri F. (2019). A phase I trial of WRSS1, a *Shigella sonnei* live oral vaccine in Bangladeshi adults and children. Hum. Vaccines Immunother..

[161] Barnoy S., Jeong K.I., Helm R.F., Suvarnapunya A.E., Ranallo R.T., Tzipori S., Venkatesan M.M. (2010). Characterization of WRSs2 and WRSs3, new second-generation virG(icsA)-based *Shigella sonnei* vaccine candidates with the potential for reduced reactogenicity. Vaccine.

[162] Frenck R.W., Baqar S., Alexander W., Dickey M., McNeal M., El-Khorazaty J., Baughman H., Hoeper A., Barnoy S., Suvarnapunya A.E. (2018). A Phase I trial to evaluate the safety and immunogenicity of WRSs2 and WRSs3; two live oral candidate vaccines against *Shigella sonnei*. Vaccine.

[163] Kim M.J., Moon Y.H., Kim H., Rho S., Shin Y.K., Song M., Walker R., Czerkinsky C., Kim D.W., Kim J.O. (2018). Cross-Protective Shigella Whole-Cell Vaccine With a Truncated O-Polysaccharide Chain. Front. Microbiol..

[164] Szijarto V., Hunyadi-Gulyas E., Emody L., Pal T., Nagy G. (2013). Cross-protection provided by live Shigella mutants lacking major antigens. Int. J. Med. Microbiol. IJMM.

[165] Herrington D.A., Van de Verg L., Formal S.B., Hale T.L., Tall B.D., Cryz S.J., Tramont E.C., Levine M.M. (1990). Studies in volunteers to evaluate candidate Shigella vaccines: Further experience with a bivalent Salmonella typhi-*Shigella sonnei* vaccine and protection conferred by previous *Shigella sonnei* disease. Vaccine.

[166] Martin P., Alaimo C. (2022). The Ongoing Journey of a Shigella Bioconjugate Vaccine. Vaccines.

[167] Phalipon A., Mulard L.A. (2022). Toward a Multivalent Synthetic Oligosaccharide-Based Conjugate Vaccine against Shigella: State-of-the-Art for a Monovalent Prototype and Challenges. Vaccines.

[168] Mo Y., Fang W., Li H., Chen J., Hu X., Wang B., Feng Z., Shi H., He Y., Huang D. (2022). Safety and Immunogenicity of a Shigella Bivalent Conjugate Vaccine (ZF0901) in 3-Month- to 5-Year-Old Children in China. Vaccines.

[169] Turbyfill K.R., Clarkson K.A., Oaks E.V., Kaminski R.W. (2022). From Concept to Clinical Product: A Brief History of the Novel Shigella Invaplex Vaccine’s Refinement and Evolution. Vaccines.

[170] Micoli F., Nakakana U.N., Berlanda Scorza F. (2022). Towards a Four-Component GMMA-Based Vaccine against Shigella. Vaccines.

[171] Camacho A.I., Irache J.M., de Souza J., Sanchez-Gomez S., Gamazo C. (2013). Nanoparticle-based vaccine for mucosal protection against *Shigella flexneri* in mice. Vaccine.

[172] Martinez-Becerra F.J., Chen X., Dickenson N.E., Choudhari S.P., Harrison K., Clements J.D., Picking W.D., Van De Verg L.L., Walker R.I., Picking W.L. (2013). Characterization of a novel fusion protein from IpaB and IpaD of *Shigella* spp. and its potential as a pan-Shigella vaccine. Infect. Immun..

[173] Chakravortty D., Pore D., Mahata N., Pal A., Chakrabarti M.K. (2011). Outer Membrane Protein A (OmpA) of *Shigella flexneri* 2a, Induces Protective Immune Response in a Mouse Model. PLoS ONE.

[174] Kim J.O., Rho S., Kim S.H., Kim H., Song H.J., Kim E.J., Kim R.Y., Kim E.H., Sinha A., Dey A. (2015). Shigella outer membrane protein PSSP-1 is broadly protective against Shigella infection. Clin. Vaccine Immunol..

[175] Barry E., Cassels F., Riddle M., Walker R., Wierzba T. (2019). Vaccines Against Shigella and Enterotoxigenic *Escherichia coli*: A summary of the 2018 VASE Conference. Vaccine.

[176] Dharmasena M.N., Osorio M., Takeda K., Stibitz S., Kopecko D.J. (2017). Stable Chromosomal Expression of *Shigella flexneri* 2a and 3a O-Antigens in the Live Salmonella Oral Vaccine Vector Ty21a. Clin. Vaccine Immunol..

[177] Barel L.A., Mulard L.A. (2019). Classical and novel strategies to develop a *Shigella* glycoconjugate vaccine: From concept to efficacy in human. Hum. Vaccin. Immunother..

[178] Robbins J.B., Shneerson R., Szu S.C. (1995). Perspective: Hypothesis: Serum IgG antibody is sufficient to confer protection against infectious diseases by inactivating the inoculum. J. Infect. Dis..

[179] Riddle M.S., Kaminski R.W., Di Paolo C., Porter C.K., Gutierrez R.L., Clarkson K.A., Weerts H.E., Duplessis C., Castellano A., Alaimo C. (2016). Safety and Immunogenicity of a Candidate Bioconjugate Vaccine against *Shigella flexneri* 2a Administered to Healthy Adults: A Single-Blind, Randomized Phase I Study. Clin. Vaccine Immunol..

[180] Cohen D., Atsmon J., Artaud C., Meron-Sudai S., Gougeon M.-L., Bialik A., Goren S., Asato V., Ariel-Cohen O., Reizis A. (2020). Safety and immunogenicity of a synthetic carbohydrate conjugate vaccine against *Shigella flexneri* 2a in healthy adult volunteers: A phase 1, dose-escalating, single-blind, randomised, placebo-controlled study. Lancet Infect. Dis..

[181] Beijing Zhifei Lvzhu Biopharmaceutical Co., Ltd. Safety Study of *S. flexneriza*-*S. sonnei* Bivalent Conjugate Vaccine in Healthy Volunteers Aged above 3 Months. https://clinicaltrials.gov/ct2/show/NCT03561181.

[182] Tribble D., Kaminski R., Cantrell J., Nelson M., Porter C., Baqar S., Williams C., Arora R., Saunders J., Ananthakrishnan M. (2010). Safety and immunogenicity of a *Shigella flexneri* 2a Invaplex 50 intranasal vaccine in adult volunteers. Vaccine.

[183] Riddle M.S., Kaminski R.W., Williams C., Porter C., Baqar S., Kordis A., Gilliland T., Lapa J., Coughlin M., Soltis C. (2011). Safety and immunogenicity of an intranasal *Shigella flexneri* 2a Invaplex 50 vaccine. Vaccine.

[184] Harro C.R., Riddle M.S., Kaminski R., Turbyfill K.R., Gormley R., Porter C., Ranallo R.T., Kordis A., Buck M., Jones A. *Shigella flexneri* 2a Invaplex 50 intranasal vaccine phase 2b challenge study. Proceedings of the Vaccines for Enteric Diseases.

[185] Gerke C., Colucci A.M., Giannelli C., Sanzone S., Vitali C.G., Sollai L., Rossi O., Martin L.B., Auerbach J., Di Cioccio V. (2015). Production of a *Shigella sonnei* Vaccine Based on Generalized Modules for Membrane Antigens (GMMA), 1790GAHB. PLoS ONE.

[186] Launay O., Lewis D.J.M., Anemona A., Loulergue P., Leahy J., Sciré A.S., Maugard A., Marchetti E., Zancan S., Huo Z. (2017). Safety Profile and Immunologic Responses of a Novel Vaccine Against *Shigella sonnei* Administered Intramuscularly, Intradermally and Intranasally: Results From Two Parallel Randomized Phase 1 Clinical Studies in Healthy Adult Volunteers in Europe. EBioMedicine.

[187] Obiero C.W., Ndiaye A.G.W., Scire A.S., Kaunyangi B.M., Marchetti E., Gone A.M., Schutte L.D., Riccucci D., Auerbach J., Saul A. (2017). A Phase 2a Randomized Study to Evaluate the Safety and Immunogenicity of the 1790GAHB Generalized Modules for Membrane Antigen Vaccine against *Shigella sonnei* Administered Intramuscularly to Adults from a Shigellosis-Endemic Country. Front. Immunol..

[188] Launay O., Ndiaye A.G.W., Conti V., Loulergue P., Sciré A.S., Landre A.M., Ferruzzi P., Nedjaai N., Schütte L.D., Auerbach J. (2019). Booster Vaccination With GVGH *Shigella sonnei* 1790GAHB GMMA Vaccine Compared to Single Vaccination in Unvaccinated Healthy European Adults: Results From a Phase 1 Clinical Trial. Front. Immunol..

[189] Kapulu M.C., Nakakana U., Sciré A.S., Sarakinou E., Conti V., Rossi O., Acquaviva A., Necchi F., Obiero C.W., Martin L.B. (2022). Complement-mediated serum bactericidal activity of antibodies elicited by the *Shigella sonnei* GMMA vaccine in adults from a shigellosis-endemic country: Exploratory analysis of a Phase 2a randomized study. Front. Immunol..

[190] Micoli F., Rossi O., Conti V., Launay O., Sciré A.S., Aruta M.G., Nakakana U.N., Marchetti E., Rappuoli R., Saul A. (2021). Antibodies Elicited by the *Shigella sonnei* GMMA Vaccine in Adults Trigger Complement-Mediated Serum Bactericidal Activity: Results From a Phase 1 Dose Escalation Trial Followed by a Booster Extension. Front. Immunol..

[191] Finco O., Rappuoli R. (2014). Designing vaccines for the twenty-first century society. Front. Immunol..

[192] MacLennan C.A., Saul A. (2014). Vaccines against poverty. Proc. Natl. Acad. Sci. USA.

[193] Shen A.K., Weiss J.M., Andrus J.K., Pecenka C., Atherly D., Taylor K., McQuestion M. (2016). Country Ownership And Gavi Transition: Comprehensive Approaches To Supporting New Vaccine Introduction. Health Aff..

[194] WHO (2015). Meeting of the Strategic Advisory Group of Experts on immunization, October 2015—conclusions and recommendations. Wkly. Epidemiol. Rec..

[195] Rogawski E.T., Liu J., Platts-Mills J.A., Kabir F., Lertsethtakarn P., Siguas M., Khan S.S., Praharaj I., Murei A., Nshama R. (2018). Use of quantitative molecular diagnostic methods to investigate the effect of enteropathogen infections on linear growth in children in low-resource settings: Longitudinal analysis of results from the MAL-ED cohort study. Lancet Glob. Health.

[196] Nasrin D., Blackwelder W.C., Sommerfelt H., Wu Y., Farag T.H., Panchalingam S., Biswas K., Saha D., Jahangir Hossain M., Sow S.O. (2021). Pathogens Associated With Linear Growth Faltering in Children With Diarrhea and Impact of Antibiotic Treatment: The Global Enteric Multicenter Study. J. Infect. Dis..

[197] CDC (2013). Antibiotic Resistance Threats in the United States. https://www.cdc.gov/drugresistance/pdf/ar.

[198] Chung The H., Rabaa M.A., Pham Thanh D., De Lappe N., Cormican M., Valcanis M., Howden B.P., Wangchuk S., Bodhidatta L., Mason C.J. (2016). South Asia as a Reservoir for the Global Spread of Ciprofloxacin-Resistant *Shigella sonnei*: A Cross-Sectional Study. PLoS Med..

[199] Pavlinac P.B., Rogawski McQuade E.T., Platts-Mills J.A., Kotloff K.L., Deal C., Giersing B.K., Isbrucker R.A., Kang G., Ma L.F., MacLennan C.A. (2022). Pivotal Shigella Vaccine Efficacy Trials-Study Design Considerations from a Shigella Vaccine Trial Design Working Group. Vaccines.

